# Serious adverse events and coping strategies of CAR-T cells in the treatment of malignant tumors

**DOI:** 10.3389/fimmu.2022.1079181

**Published:** 2022-12-08

**Authors:** Xiujin Chen, Peng Li, Bin Tian, Xin Kang

**Affiliations:** Department of Orthopedics, Honghui Hospital, Xi’an Jiaotong University, Xi’an, China

**Keywords:** CAR-T, serious adverse events, lymphoma, leukemia, multiple myeloma, solid tumor, CRS, ICANS

## Abstract

Chimeric antigen receptor T (CAR-T) cells technology has been successfully used in the treatment of B cell-derived hematological tumors and multiple myeloma. CAR-T cells are also being studied in a variety of solid tumors. Current clinical reports on CAR-T cells in the treatment of malignant tumors are abundant. The tumor-killing activity of CAR-T cells and the unique adverse effects of CAR-T cells have been confirmed by many studies. There is evidence that serious adverse events can be life-threatening. CAR-T cells therapy is increasingly used in clinical settings, so it is important to pay attention to its serious adverse events. In this review, we summarized the serious adverse events of CAR-T cells in the treatment of malignant tumors by reading literature and searching relevant clinical studies, and discussed the management and treatment of serious adverse events in an effort to provide theoretical support for clinicians who deal with such patients.

## 1 Introduction

Immunotherapy has become a mainstay of cancer treatment, in addition to standard surgery, chemotherapy and radiation (1). The discovery of tumor-mediated immunosuppression and its relationship to malignant tumor progression laid the foundation for the application of T cells therapy strategies (2). Thus, gene-edited T cells immunotherapy has been rapidly developed in recent years. Chimeric antigen receptor T cells (CAR-T) are genetically reprogrammed T cells that express antibody fragments that bind specifically to tumor-surface antigens (3). The mechanism of tumor killing is that CAR-T cells bind to tumor antigens and induce a potent antitumor immune response (4, 5). Recently, CD19-targeting CAR-T cells have shown significant efficacy in patients with relapsed/refractory (R/R) CD19+ B cell malignancies (6–10). Targeting BCMA or CD22 CAR-T cells has also demonstrated potent antitumor activity in clinical studies of multiple myeloma and acute lymphoblastic leukemia (11–15). Moreover, CAR-T cells are being studied in solid tumors, although they have shown limited efficacy so far (16–21).

Immune system activation-related toxicities have been shown in clinical studies involving CAR-T cells (22). The toxic symptoms experienced after CAR-T cells therapy are mainly caused by cytokine release syndrome (CRS) and immune effector cell associated neurotoxicity (ICANS) (23). Currently, although the safety profile of CAR-T cells therapy is generally acceptable, the incidence of serious adverse events (SAEs) is high among clinical trials using CAR-T cells (24–26). Therefore, it is crucial to systematically evaluate the toxicity characteristics and life-threatening potential of CAR-T cells therapies. In this article, we downloaded CAR-T cells related clinical study data from the Clinical Trials Database (www.clinicaltrials.gov). In combination with published clinical studies, the clinical manifestations of SAEs of CAR-T cells in the treatment of solid and hematological tumors were summarized. Finally, the management and treatment measures of SAEs were discussed to lay a theoretical foundation for the better application of CAR-T cells in clinical practice.

## 2 Clinical presentation of SAEs associated with CAR-T cells therapy

**Figure 1:**
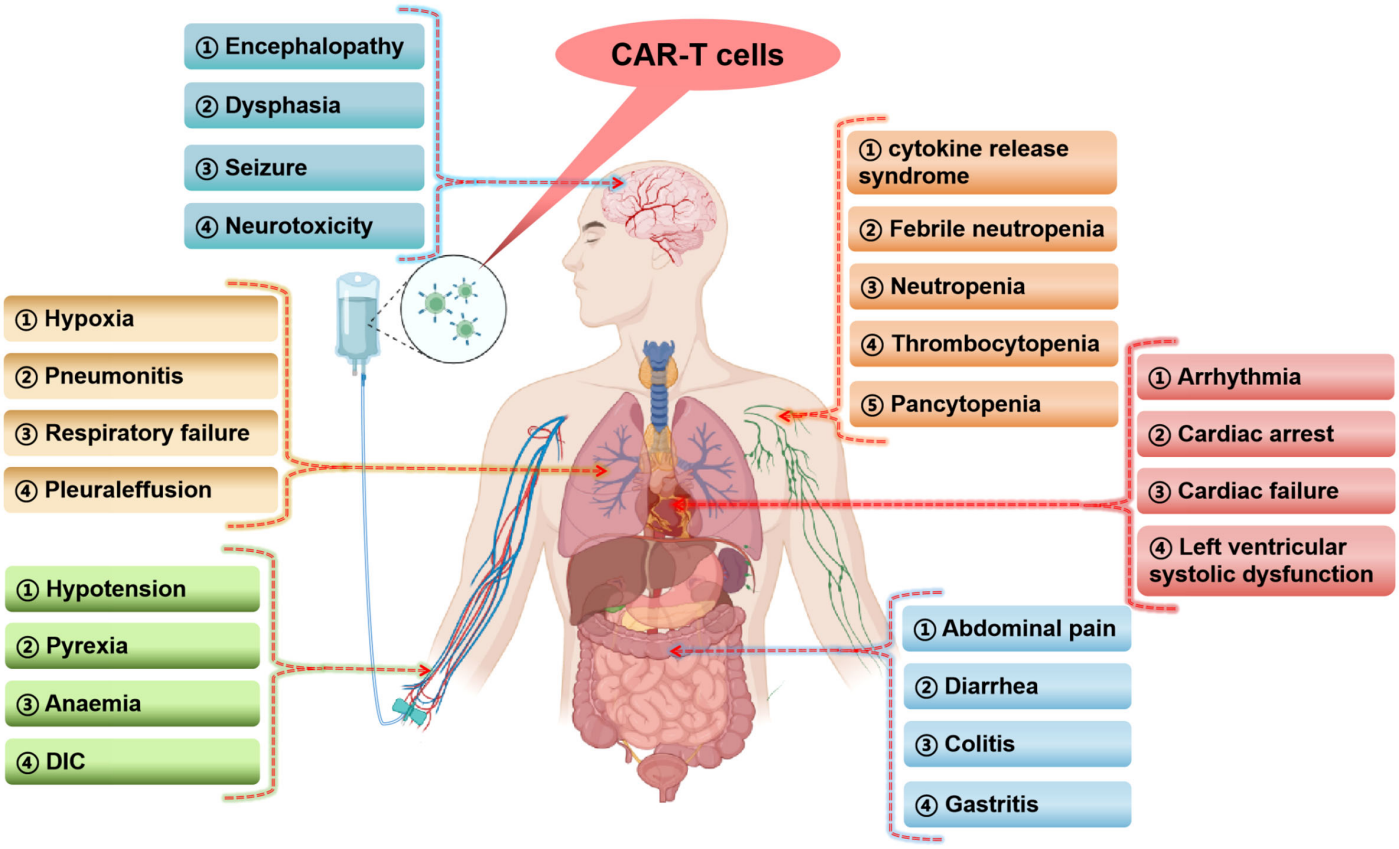
Occurrence of serious adverse events in various human systems in CAR-T cells clinical studies (The figure is produced using the BioRender online graphics website). DIC, disseminated intravascular coagulation.

Clinicians should be aware of the serious and potentially fatal toxicity associated with CAR-T cells therapy, although they hold promise for the treatment of certain cancers (27). In this study, 24 clinical studies (1208 cases) in hematological tumors and 7 clinical studies (92 cases) in solid tumors were downloaded from the clinical trial database (www.clinicaltrials.gov), and the trial results data were available for all the downloaded clinical studies (**Table 1**–**4**). In addition, the data of SAEs from the included clinical studies were analyzed, and the occurrence of SAEs in the treatment of malignant tumors with CAR-T cells was systematically summarized in combination with the relevant published literature. Numerous clinical studies have shown that CAR-T cells can cause SAEs in the treatment of both hematological and solid tumors (**Figure 1**). The SAEs can affect any organ system of the body, and can develop into multiple organ failure in severe cases, endangering life.

**Table 1:** The incidence of clinically serious adverse events of CAR-T in hematological tumors.

NCT Number	Conditions	Interventions	Characteristics	countrys	Adverse event assessment criteria	Enrollment	All-Cause Mortality(n/Total)	Serious adverse events(n/Total)	Other (Not Including Serious) Adverse Events(n/Total)
NCT03958656	Myeloma;Multiple Myeloma	Anti-Signaling;lymphocytic activation molecule F7 (SLAMF7);chimeric antigen receptor(CAR) T cells	Phase 1	United States	CTCAE v5.0	10	0/10	3/10	10/10
NCT03287804	Multiple Myeloma	AUTO2	Phase 1 Phase 2	United Kingdom	CTCAE v4.0	11	8/11	6/11	11/11
NCT03289455	B-cell Acute Lymphoblastic Leukemia	AUTO3 (CD19/22 CAR-T cells	Phase 1 Phase 2	United Kingdom	CTCAE v5.0	15	9/15	6/15	15/15
NCT00924326	Primary Mediastinal B-cell Lymphoma; Diffuse, Large B-cell; Lymphoma	Anti-CD19-CAR-T cells	Phase 1 Phase 2	United States	CTCAE 3.0	46	2/46	29/46	46/46
NCT03019055	Lymphoma;Non-Hodgkin,Lymphoma, B-Cell;Small Lymphocytic Lymphoma	CAR-20/19- T cells	Phase 1	United States	CTCAE v4.0	22	0/22	22/22	22/22
NCT02659943	Lymphoma;B-Cell,Lymphoma, Non-hodgkins	Anti-CD19-CAR-T cells	Phase 1	United States	CTCAE v5.0	21	0/21	17/21	21/21
NCT02794246	Multiple Myeloma	Anti-CD19-CAR-T cells	Phase 2	United States	CTCAE v4.03	6	0/6	2/6	1/6
NCT01747486	Relapsed or Refractory CLL or SLL	Anti-CD19-CAR-T cells	Phase 2	United States	CTCAE v4.0	42	12/42	32/42	35/42
NCT02215967	Myeloma-Multiple Myeloma	Anti- BCMA-CAR-T cells	Phase 1	United States	CTCAE 4.0	26	0/26	13/26	26/26
NCT02535364	Acute Lymphoblastic Leukemia	Anti-CD19-CAR-T cells	Phase 2	United States	CTCAE v4.0	38	24/38	23/38	38/38
NCT01593696	B Cell Lymphoma, Leukemia	Anti-CD19-CAR-T cells	Phase 1	United States	CTCAE v4.0	53	29/53	14/53	53/53
NCT01593696	Recurrent Plasma Cell Myeloma	BCMA CAR-T Cells	Phase 1	United States	CTCAE v4.0	25	7/25	21/25	25/25
NCT01593696	Lymphoma;Lymphoma, Large B-Cell, Diffuse;Lymphoma, Extranodal NK-T Cell;Lymphoma, T-Cell,Peripheral	Anti-CD30 CAR-T Cells	Phase 1	United States	CTCAE v5.0	22	0/22	10/22	22/22
NCT03318861	Relapsed/Refractory Multiple Myeloma	BCMA-CAR-T cells(KITE-585)	Phase 1	United States	CTCAE v 4.03	14	7/14	1/14	14/14
NCT01593696	ALL;B Cell Lymphoma;Leukemia;Large CellLymphoma;Non-Hodgkin Lymphoma	Anti-CD19-CAR-T cells	Phase 1	United States	CTCAE v 4.0	53	29/53	14/53	53/53
NCT03624036	Relapsed/Refractory Chronic Lymphocytic Leukemia and Relapsed/ Refractory Small Lymphocytic Lymphoma	Anti-CD19-CAR-T cells(KTE-X19)	Phase 1	United States	CTCAE v 5.0	16	3/16	7/16	16/16
NCT02030847	Patients With B Cell ALL, Relapsed or Refractory	CD19-CAR-T	Phase 2	United States	CTCAE v 4.0	30	30/30	30/30	30/30
NCT02614066	Relapsed/Refractory Bprecursor Acute Lymphoblastic Leukemia	Anti-CD19 CAR-T Cells	Phase 1 Phase 2	United States	CTCAE v 4.0	125	65/125	80/125	125/125
NCT03761056	B-cell Lymphoma	anti-CD19 CAR-T	Phase 2	United States,Australia and France	CTCAE v5.0	40	6/40	18/40	40/40
NCT01865617	Recurrent Adult Acute Lymphoblastic Leukemia;Recurrent Chronic Lymphocytic Leukemia;Recurrent Diffuse Large B-Cell Lymphoma Recurrent Mantle Cell Lymphoma	anti-CD19 CAR-T	Phase 1 Phase 2	United States	CTCAE v 4.0	197	115/197	189/197	196/197
NCT02348216	B-Cell Lymphoma;Transformed Follicular Lymphoma (TFL)	anti-CD19 CAR-T	Phase 1 Phase 2	United States	CTCAE v 4.0	292	115/292	153/292	292/292
NCT02926833	Refractory Diffuse Large B Cell Lymphoma	anti-CD19 CAR-T	Phase 1 Phase 2	United States	CTCAE v 4.0	34	11/34	23/34	34/34
NCT02706405	B Cell Lymphoma	anti-CD19 CAR-T	Phase 1	United States	CTCAE v 4.03	29	13/29	19/29	29/29
NCT03568461	Follicular Lymphoma	anti-CD19 CAR-T	Phase 2	United States	CTCAE v 4.03	97	7/97	42/97	94/97

All clinicaltrials can be downloaded from www.clinicaltrials.gov (accessed October 02, 2022).

**Table 2:** Summary of clinical serious adverse events of CAR-T in hematological tumors(Patients Number/symptom).

NCT Number(Patients Number)	General complications	Infections and infestations	Cardiac complications	Nervous system complications	Immune system complications	Blood and lymphatic system complications	Respiratory, thoracic and mediastinal complications	Gastrointestinal complications	Vascular complications
NCT03287804 (11)	2/Pyrexia	1/Lung infection	1/Acute myocardial infarction	1/Hedache			1/Dyspnoea		
NCT03289455 (15)	1/Pyrexia	1/Cellulitis		1/Encephalopathy;1/Seizure		3/Anaemia;3/Neutropenia; 3/Thrombocytopenia;2/Febrile neutropenia			
NCT00924326 (46)	3/Fever	1/Pneumonia	2/Arrhythmia. Supraventricular tachycardia;1/Supraventricular and nodal arrhythmia;1/Atrial fibrillation;1/Left ventricular systolic dysfunction	12/Speech impairment; 10/Confusion; 9/Somnolence, depressed level of consciousness; 4/Neuropathy,motor; 2/Seizure; 2/Ataxia;2/Cognitive disturbance; 1/CNS cerebrovascular ischemia;1/Encephalopathy		6/Febrile neutropenia; 1/Lymphopenia	4/Hypoxia; 2/Dyspnea	1/Colitis;2/Dysphagia	5/Hypotension;2/Thrombosis
NCT03338972 (25)	11/fever	1/lung infection;1/upper respiratory infection			1/CRS	8/febrile neutropenia; 2/neutropenic fever		1/nausea	1:hypotension
NCT02535364 (38)	1/Asthenia; 1/Pyrexia	2/Sepsis;1/Bacteraemia	1/Atrial fibrillation; 1/Myocardial infarction	8/Encephalopathy; 5/Brain oedema; 2/Seizure	8/CRS	1/Febrile neutropenia		1/Neutropenic colitis;1/Abdominal pain	
NCT03049449 (22)	2/Fever	3/Sepsis	3/Sinus tachycardia	1/Encephalopathy		1/Anemia	1/Dyspnea; 1/Hypoxia	1/Diarrhea; 1/Nausea	4/Hypotension
NCT03318861(14)	1/Chest pain						1/Hypoxia		
NCT01593696(53)	3/Fever		3/Sinus tachycardia;2/Left ventricular systolic dysfunction; 1/Cardiac arrest; 1/Heart failure	4/Nervous system complications; 2/Seizure; 1/Dysphasia; 1/Headache; 1/Hydrocephalus; 1/Somnolence	9/CRS		2/Hypoxia; 1/Pulmonary edema; 1/Respiratory failure		2/Hypotension;1/Hypertension
NCT03624036(16)	2/Pyrexia; 1/Malaise	1/Sepsis; 1/Systemic candida	1/Tachycardia	1/Aphasia; 1/Confusional state	4/CRS			1/Abdominal pain	3/Hypotension;1/Embolism
NCT02030847(30)		3/Sepsis;2/Pneumonia;1/Meningitis;1/Staphylococcal infection		1/Haemorrhage intracranial; 1/Headache;1/Seizure	21/CRS	1/Febrile neutropenia	1/Hypoxia	1/Constipation	
NCT02614066(125)	20/Pyrexia;2:Fatigue; 1:Chills; 1:Multiple organ dysfunction syndrome;1:Face oedema	9/Bacteraemia;7/Sepsis;6/Pneumonia;1/Cellulitis	9/tachycardia;1/Cardiomyopathy	15/Encephalopathy;7/Aphasia;5/Seizure;2/Cerebrovascular accident;1/Immune effector cell-associated neurotoxicity syndrome;1/Brain oedema; 1/Facial paralysis 1/Headache	1/Drug hypersensitivity;1/Graft versus host disease;	6/Febrile neutropenia; 2/Pancytopenia;2/Disseminated intravascular coagulation; 1/Cytopenia; 1/Neutropenia	13/Hypoxia;5:Respiratory failure; 4:ARDS;3/Dyspnoea;1/Pulmonary embolism	2/Colitis;2/Ileus;1/Diarrhoea;1/Gastritis	31/Hypotension;1/Hypertension;1/Shock
NCT03019055(22)	1/Fever;1/Multi-organ failure	1/Upper respiratory infection		1/Nervous system complications - Other, specify	5/CRS	4/Blood and lymphatic system complications; 1/Febrile neutropenia	1/Pleural effusion; 1/Pneumonitis	1/Diarrhea	
NCT03761056 (40)	3/Pyrexia;2/Non-cardiac chest pain	3/infection;1/Covid-19;1/Covid-19 pneumonia;1/Cytomegalovirus infection reactivation	1/Atrial fibrillation; 1/Sinus bradycardia; 1/Supraventricular tachycardia	5/Encephalopathy;1/Neurotoxicity;1/Dysarthria;1/Memory impairment; 1/Haemorrhage intracranial		1/Anaemia;1/Neutropenia	1/Acute pulmonary oedema	1/Abdominal pain	1/Hypertension;1/Hypotension
NCT01865617 (195)	17/Fever;3/Multi-organ failure	9/Infections and infestations-Other, specify;6/Lung infection; 3/Sepsis	3/Atrial fibrillation; 3/Sinus tachycardia; 2/Cardiac arrest; 2/Heart failure; 2/Left ventricular systolic dysfunction	18/Encephalopathy;4/Seizure; 4/Depressed level of consciousness;2/Edema cerebral;2/Nervous system complications;1/Dysphasia	41/CRS	132/Febrile neutropenia; 2/Disseminated intravascular coagulation;	8/Respiratory failure;6/Hypoxia;3/Pleural effusion; 3/Pulmonary edema;2/ARDS;1/Dyspnea	2/Abdominal pain;2/Nausea	34/Hypotension
NCT02659943(21)	1/Fever	1/Lung infection	1/Cardiac arrest; 1/Sinus tachycardia	3/Syncope;1/Encephalopathy;1/Tremor		1/Anemia;1/Neutrophil count decreased	3/Hypoxia	2/Diarrhea;1/Abdominal pain; 1/Ileus	6/Hypotension
NCT02348216 (292)	25/Pyrexia	7/Lung infection; 3/Bacteraemia;2/Adenovirus infection;2/Covid-19; 1/Covid-19 pneumonia	4/Atrial fibrillation; 4/Cardiac arrest; 2/Atrial flutter; 2/Cardiac failure	29/Encephalopathy;10/Aphasia;8/Somnolence;5/Seizure;3/Headache;3/Syncope;2/Depressed level of consciousness; 2/Haemorrhage intracranial; 1/Immune effector cell-associated neurotoxicity syndrome;		12/Febrile neutropenia; 5/Neutropenia;5/Pancytopenia;2/Thrombocytopenia; 2/Bone marrow failure	7/Hypoxia;2/Acute respiratory failure; 2/Pleural effusion	3/Abdominal pain;3/Pancreatitis;2/Dysphagia	13/Hypotension
NCT02926833 (34)	3/Pyrexia;1/Multiple organ dysfunction syndrome;1/Localised oedema	1/Lung infection; 1/Sepsis	1/Supraventricular tachycardia	10/Encephalopathy;2/Seizure;1/Aphasia	1/Haemophagocytic lymphohistiocytosis	2/Anaemia;1/Neutropenia;1/Febrile neutropenia	3/Hypoxia;1/Respiratory failure; 1/Pleural effusion	1/Abdominal pain;1/Diarrhoea;1/Obstruction gastric	2/Hypotension
NCT02215967(25)	2/Fever	2/Lung infection; 2/Upper respiratory infection	4/Sinus tachycardia; 1/Supraventricular tachycardia	1/Encephalopathy		1/Disseminated intravascular coagulation	6/Dyspnea;3/Hypoxia	2/Diarrhea	6/Hypotension
NCT02706405(29)	5/Fever;1/Multi-organ failure	1/Bacteremia	2/Sinus tachycardia	2/Encephalopathy;1/Somnolence	9/CRS	3/Febrile neutropenia	1/Dyspnea;1/Pleural effusion	2/Abdominal pain;1/Duodenal hemorrhage	1/Hypotension
NCT03958656(10)	1/Fever		2/Sinus tachycardia		1/CRS				
NCT03568461(97)	3/Pyrexia	8/Pneumonia;6/encephalitis;1/Bacteraemia;1/COVID-19;1/COVID-19 pneumonia; 1/Lower respiratory tract infection;1/Sepsis	1/Ventricular fibrillation	2/Encephalopathy;1/Headache;1/Immune effector cell-associated neurotoxicity syndrome;1/Syncope	19/CRS;1/Graft versushost disease in gastrointestinal tract	6/Febrile neutropenia; 2/Neutropenia;1/Anaemia	2/Pleural effusion; 1/Acute respiratory failure;1/Dyspnoea;1/Pneumothorax	1/Gastrointestinal ulcer;1/Nausea;1/Vomiting;1/Stomatitis	
NCT02794246(6)		1/Upper respiratory infection			1/CRS				
NCT01747486(42)	10/Pyrexia;1/Fatigue	2/Pneumonia;2/Upper respiratory tract infection; 1/Sepsis		1/Encephalopathy;1/Syncope	18/CRS	8/Febrile Neutropenia	1/Hypoxia;1/Pneumonitis;1/Pulmonary oedema	1/Abdominal Pain;1/Diarrhoea	

All clinicaltrials can be downloaded from www.clinicaltrials.gov (accessed October 02, 2022).

**Table 3:** The incidence of clinical serious adverse events of CAR-T in solid tumors.

NCT Number	Conditions	Interventions	Characteristics	Country	Adverse event assessment criteria	Enrollment/n	All-Cause Mortality(n/Total)	Serious adverse events(n/Total)	Other (Not Including Serious) Adverse Events(n/Total)
NCT02664363	Glioblastoma;Gliosarcoma	EGFRvIII CAR-T cells	Phase 1	United States	CTCAE v5.0	3	3/3	1/3	3/3
NCT03330834	Advanced Lung Cancer	PD-L1 CAR-T cells	Phase 1	China	CTCAE v4.0	1	1/1	1/1	1/1
NCT01454596	Malignant Glioma; Glioblastoma; Brain Cancer; Gliosarcoma	EGFRvIII CAR-T cells	Phase 1 Phase 2	United States	CTCAE v4.0	18	1/18	2/18	18/18
NCT01583686	Cervical Cancer; Pancreatic Cancer; Ovarian Cancer; Mesothelioma;Lung Cancer	Anti-mesothelin CAR-T cells	Phase 1 Phase 2	United States	CTCAE v4.0	15	1/15	5/15	15/15
NCT01218867	Metastatic Cancer; Metastatic Melanoma; Renal Cancer	Anti-VEGFR2 CAR-T cells	Phase 1 Phase 2	United States	CTCAE v3.0	22	1/22	5/22	21/22
NCT02761915	Relapsed or Refractory Neuroblastoma	Genetic/1RG-CAR-T cells	Phase 1	United Kingdom	CTCAE v4.0	12	6/12	5/12	12/12
NCT02706392	Hematopoietic and Lymphoid Cell Neoplasm; Malignant Solid Neoplasm; Metastatic Lung Non-Small Cell Carcinoma; Metastatic Triple-Negative Breast Carcinoma; Recurrent Acute Lymphoblastic Leukemia; Recurrent Mantle Cell Lymphoma; Refractory Chronic Lymphocytic Leukemia	ROR1 CAR-T cells	Phase 1	United States	CTCAE v4.0	21	12/21	17/21	21/21

All clinicaltrials can be downloaded from www.clinicaltrials.gov (accessed October 02, 2022).

**Table 4:** Summary of clinical serious adverse events of CAR-T in solid tumors(Patients Number/symptom).

NCT Number(Patients Number)	General complications	Infections and infestations	Nervous system complications	Immune system complications	Blood and lymphatic system complications	Respiratory, thoracic and mediastinal complications	Gastrointestinal complications	Vascular complications
NCT03330834(1)						1/1interstitial pneumonia disease		
NCT02664363(3)	1/Generalized muscle weakness		1/Confusion					
NCT01583686(15)					1/Anemia 1/Platelet count decreased;2/Lymphocyte count decreased	1/Hypoxia	1/Constipation	
NCT01218867(22)	1/Pain;3/ALT, SGPT (serum glutamic pyruvic transaminase);3/AST, SGOT (serum glutamic oxaloacetic transaminase);3/Bilirubin (hyperbilirubinemia)	1/Infection				2/Hypoxia	1/Nausea;1/Vomiting	
NCT01454596(18)	1/Multi-organ failure					1/Dyspnea (shortness of breath);1/Hypoxia		
NCT02706392(21)	13/Fever 1/Non-cardiac chest pain;1/Myalgia		1/Encephalopathy	3/CRS	3/Febrile neutropenia	2/Dyspnea 3/Hypoxia 1/Respiratory failure		3/Hypotension
NCT02761915(12)	1/Pain;5/Pyrexia	1/Post procedural cellulitis;1/Pseudomonal bacteraemia;1/Pseudomonal sepsis;1/Urinary tract infection			1/Febrile neutropenia;	1/Laryngeal haemorrhage		

All clinicaltrials can be downloaded from www.clinicaltrials.gov (accessed October 02, 2022).

### 2.1 SAEs of CAR-T cells in the treatment of hematological tumors

#### 2.1.1 Immune system toxicities

This study found that 141 patients (11.67%) had immune system SAEs, and the incidence of SAEs from high to low was the CRS (137 cases), graft versus host disease (2 cases), etc (**Table 2**). As a result of the high production of cytokines during CAR-T cells therapy, CRS is the most common SAEs of immune system (28). It was found that 128 cytokines may be closely related to CRS, among which IL6, IFN-γ, TNF-α, ICAM-1, VCAM-1, VEGFA and other important factors may be the key factors to predict CRS (29). Additionally, it causes SAEs throughout the body in a variety of systems (30). Cytokines are a double-edged sword in the process of CAR-T cells therapy, which can stimulate immune cells to kill tumor cells while also causing damage to normal organs of the body (31, 32).

Z. Ying et al. (33)conducted a meta-analysis involving 27 studies (1687 patients) to evaluate the safety of CD19-targeted CAR-T cells in patients with diffuse large B-cell lymphoma (DLBCL). Severe CRS and severe neurotoxicity were found in 6% (95%CI: 3-10%) and 16% (95%CI: 10-24%), respectively. Moreover, studies have shown that neurological SAEs are associated with CRS (34, 35). This suggests that CRS may contribute to neurological adverse events. Furthermore, M. Shao et al. (36) retrospectively analyzed the adverse events of 37 R/R MM patients treated with BCMA-targeted CAR-T cells. All of the 37 patients had CRS, and 34 (91%) had at least one coagulation parameter abnormality. The values of coagulation parameters were positively correlated with the severity of CRS, as well as with the levels of cytokines such as IL-6, IL-10 and IFN-γ. The findings suggest that these factors may play an important role in CRS-related coagulopathy as well as a connection between coagulopathy and CRS. In addition, J. Zhou et al. (37) retrospectively analyzed 133 patients with R/R lymphoma who received CAR-T cells therapy. Studies have found that severe neutropenia, anemia, and thrombocytopenia frequently occur after CAR-T cells infusion. Further studies found that both neutropenia and severe thrombocytopenia in severe patients were associated with the incidence of CRS and the levels of associated inflammatory factors. The above studies all reflect that CRS is an adverse events and a initiating factor causing various SAEs.

#### 2.1.2 Nervous system toxicities

In this study, 244 patients (20.20%) developed nervous system SAEs. The incidence of clinical symptoms from high to low was encephalopathy (94 cases), speech impairment (33 cases), seizure (24 cases), somnolence (20 cases), confusion (11 cases), syncope (8 cases), and brain oedema (8 cases), headache (8 cases), etc (**Table 2**). The most common life-threatening neurological adverse event is encephalopathy, probably due to the significant effects of CAR-T cells on cerebral vessels. Secondly, the high incidence of severe speech complications found in this study suggests that the language center may also be an easy target for CAR-T cells. Seizures are also very common, indicating that CAR-T cells disrupt brain neuronal electrical activity.

Neurotoxicity caused by CAR-T cells, also known as ICANS, is the primary cause of these complications (38). Similarly, studies have demonstrated that the most common ICANS with CAR-T cells include encephalopathy, headache, tremor, dizziness, aphasia, delirium, insomnia, and anxiety (39, 40). L. Lv et al. (41)explored the safety of CAR-T cells for central nervous system lymphoma (CNSL). A total of 63 patients were included in 8 studies in the meta-analysis, and the incidence of grade 3 or above neurotoxicity was found to be 12%. Besides, A. Gajra et al. (42) investigated adverse neurologic events associated with CAR-T cells therapy in patients with R/R large B-cell lymphoma. There are a lot of neurologic adverse events associated with CAR-T cells therapy in the real world, which is a testament to the truthfulness of clinical trial reports. Although real data on CAR-T cells-associated neurotoxicity are limited, one study found an inverse association between grade 3-4 neurotoxicity and OS (43). According to these studies, neurological dysfunction is universal and important in the clinical application of CAR-T cells therapy.

#### 2.1.3 Respiratory, thoracic and mediastinal toxicities

In this study, 103 patients (8.53%) developed respiratory, thoracic and mediastinal SAEs. The incidence of clinical symptoms from high to low were hypoxia (45 cases), respiratory failure (18 cases), dyspnea (12 cases), pleural effusion (10 cases), pulmonary edema (6 cases), ARDS (6 cases), pneumonitis (2 cases), etc (**Table 2**). The most common SAEs of the respiratory system is hypoxemia, and the disease can progress to respiratory failure. Common co-symptoms are dyspnea, pleural effusion, pulmonary edema, ARDS, and pneumonia.

Researchers have found that respiratory SAEs are a leading cause of death associated with CAR-T cells therapy. J. Pan et al. (44) evaluated the safety of anti-CD7 CAR-T cells in 20 patients with R/R T cells acute lymphoblastic leukemia (NCT04689659). The results of the study found that all adverse events were reversible, except for one patient who died from a related fungal pneumonia. Similarly, in the study of R. Benjamin et al. (45), two treatment-related deaths occurred. One was caused by neutropenic sepsis complicated by CRS, and the other by pulmonary hemorrhage with persistent cytopenia. K. Rejeski et al. (46) described the clinical course of a 59-year-old patient with R/R large B-cell lymphoma who received Axicabtagene-Ciloleucel. Severe pneumonia eventually leads to respiratory failure and death. Furthermore, respiratory adverse events may be affected by CRS. A. Goldman et al. (47) retrospectively analyzed adverse events in 2657 patients who received CD19-targeted CAR-T cells therapy. Cardiopulmonary adverse events occurred in 546 patients (20.5%). Ultimately, the mortality rate for cardiopulmonary adverse events was 30.9%. Studies have shown associations between CAR-T cells and various cardiopulmonary adverse events, including rapid respiratory failure, hypoxemia, arrhythmias, cardiomyopathy, pericardial and pleural diseases. In addition, the overlapping reports of cardiopulmonary adverse events and CRS were found in 68.3% of the cases. CRS may also be involved in the pathogenesis of severe cardiopulmonary adverse events, which should be considered in the multidisciplinary evaluation and monitoring of CAR-T cells recipients.

#### 2.1.4 Cardiovascular toxicities

In this study, 116 patients (9.60%) had vascular SAEs, and the main clinical SAEs were hypotension (109 cases), thrombosis (3 cases), hypertension (3 cases), etc (**Table 2**). 68 patients (5.63%) had cardiac SAEs. The incidence of SAEs from high to low are sinus tachycardia (28 cases), atrial fibrillation (10 cases), cardiac arrest (8 cases), and supraventricular fibrillation tachycardia (5 cases), left ventricular systolic dysfunction (5 cases), heart failure (5 cases), myocardial dysfunction (2 cases), etc (**Table 2**). Studies have found that the main SAEs of vascular complications is hypotension, the pathogenesis may be due to the occurrence of inflammation in the body produces a large number of inflammatory cytokines released into the blood, resulting in peripheral vascular dilatation (48, 49). Arrhythmias occur in the cardiovascular system to compensate for hypotension, so the most common arrhythmias are sinus tachycardia and atrial fibrillation. Severe arrhythmias can progress to cardiac arrest and eventually lead to heart failure (50). In addition, symptoms of left ventricular dysfunction have been seen in clinical studies (48, 51). Therefore, the occurrence of adverse cardiovascular events may be due to the massive cytokine release during CAR-T cells therapy.

Cardiovascular toxicity is not uncommon in patients receiving CAR-T cells therapy (52). Adam Goldman et al. (47) found that the occurrence of tachyarrhythmia was a major adverse effect of the heart. Atrial fibrillation is the main tachyarrhythmia, followed by ventricular arrhythmia. Studies have also shown an association between CAR-T cells and symptoms such as tachyarrhythmia, cardiomyopathy, pericardial and pleural disease. Additionally, 10-30% of patients also exhibit decreased left ventricular ejection function (48). R. M. Alvi et al. (53) also reported a new reduction in ejection fraction in 8 of 137 patients, 5 patients also experienced arrhythmias, and 6 patients experienced cardiovascular death. To examine cardiovascular adverse events associated with CAR-T cells, A. Guha et al. (54) used the U.S. Food and Drug Administration Adverse Event Reporting System (FAERS) to observe 996 cases in which the most commonly reported cardiovascular adverse event was arrhythmia (77.6%). This was followed by heart failure (14.3%) and myocardial infarction (0.5%). Cardiovascular adverse events associated with CAR-T cells therapy were also associated with higher mortality. Therefore, the use of CAR-T cells in tumor therapy should be vigilant for cardiovascular events.

#### 2.1.5 Gastrointestinal toxicities

In this study, 48 patients (3.97%) had gastrointestinal SAEs. The incidence of SAEs from high to low were abdominal pain (13 cases), diarrhea (9 cases), nausea (5 cases), colitis (4 cases), dysphagia (4 cases), pancreatitis (3 cases), etc (**Table 2**). The adverse events of CAR-T cells on the digestive system are relatively less, and SAEs are mainly caused by gastroenteritis leading to abdominal pain, diarrhea and other clinical manifestations. A small number of adverse events of pancreatitis were also observed. These results suggest that CAR-T cells may be mainly through its cytokines acting on gastrointestinal mucosa, leading to impaired barrier function and the progression of mucositis (55). The incidence of SAEs in the digestive system is significantly less than that in the nervous, immune, cardiovascular and respiratory systems. Moreover, the severity of adverse effects is relatively mild, and no serious life-threatening adverse events have been reported.

#### 2.1.6 Infections and infestations

Infection-related SAEs occurred in 116 patients (9.60%). The incidence of SAEs from high to low were lung infection (33 cases), upper respiratory infection(7 cases), sepsis (22 cases), bacteraemia(15 cases), Covid-19(4 cases), and Covid-19 pneumonia(3 cases), etc (**Table 2**). The most common infection is a respiratory tract infection, which can involve the lungs in severe cases. Telli Dizman et al. (56) conducted a systematic review and meta-analysis of the incidence of severe infections in hematological malignancies treated with CAR-T cells. The severe infection rate was 16.2%, with the respiratory tract being the most common site of infection. This also confirms the above views. The common pathogen is bacteria, but it can also be seen in clinical studies of COVID-19 infection. Besides, severe bacteremia and septicemia are often seen. The immune barrier function may be impaired during CAR-T cells therapy, allowing opportunistic pathogens to flourish (57).

Most infections after CAR-T cells therapy occur after neutropenia and/or severe CRS, indicating a greater degree of immune impairment (58, 59). Furthermore, most CAR-T cells recipients had previously received other antitumor therapies, including autologous and allogeneic hematopoietic cell transplants. Preexisting cytopenia and hypogammaglobulinemia increase the likelihood of infection (60, 61). The occurrence of CRS co-infection may lead to a greater impact on the body, which may not respond well to antimicrobial therapy. In the study conducted by J. A. Hill et al. (58), 80% of patients had their first infection within the first 10 days after CAR-T cells infusion, mainly with gram-negative bacterial infections. Besides, 42% of patients had predominantly viral infections within 30 days of infusion, including respiratory viral infections and cytomegaloviremia and pneumonia. Later infection may reflect a state of immunoglobulin deficiency and lymphocytopenia (58). These studies suggest that serious infection-related adverse events associated with CAR-T cells therapy are not only related to CRS, but also to the patient’s immunocompromised physical condition, posing a serious threat to patient health.

#### 2.1.7 Blood and lymphatic system toxicities

Blood and lymphatic system SAEs were found in 228 patients (18.87%). The incidence of SAEs from high to low is febrile neutropenia (187 cases), neutropenia (12 cases), anaemia (9 cases), pancytopenia (8 cases), thrombocytopenia (5 cases), and disseminated intravascular coagulation (DIC) (5 cases), etc (**Table 2**). The most common SAEs of hemolymph system is neutropenia. As an important immune cell, neutrophils play an important role in preventing the invasion of pathogenic microorganisms. However, neutrophil depletion during CAR-T cells treatment may account for the susceptibility of the body to infection-related diseases. Besides, the study found that patients also had a decrease in various blood cells and platelets (62), which indicates that the blood system may be seriously damaged during the treatment.

When injected into the bloodstream to kill tumors, CAR-T cells have been shown to be hemotoxic (62). L. Wang et al. (63) retrospectively studied the characteristics and risk factors of new-onset severe cytopenia after CAR-T cells infusion in 76 patients with R/R acute lymphoblastic leukemia. A high incidence of new severe cytopenia was found, including severe neutropenia (56,70%), severe anemia (66,53%), and severe thrombocytopenia (64,48%). The study also found that people with higher levels of CRS had higher incidence and longer duration of severe cytopenia. Multivariate analysis showed that the occurrence of CRS and higher grade of CRS were risk factors for prolonged hematotoxicity. These observations lead to the conclusion that the occurrence of CRS is associated with the incidence of severe cytopenia, suggesting that CRS may be a direct or indirect cause of hemotoxicity.

#### 2.1.8 General toxicities

General SAEs occurred in 133 patients (11.01%). The incidence of SAEs from high to low was pyrexia (116 cases), multi-organ failure (7 cases), fatigue (3 cases), etc (**Table 2**). The most common adverse effect of the body is pyrexia, which is mainly caused by the massive release of inflammatory factors into the blood during CRS, but the possibility of subsequent infection after the immune system is compromised cannot be ruled out (57). Therefore, it is difficult to distinguish CRS or infection from fever alone during CAR-T cell therapy.

### 2.2 SAEs of CAR-T in the treatment of solid tumors

**Figure 2:**
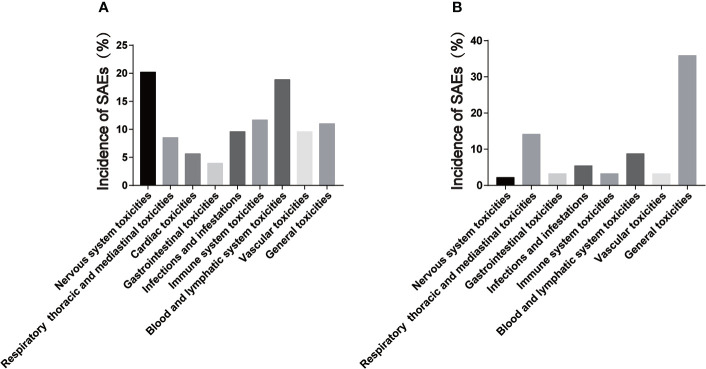
Incidence of serious adverse events of CAR-T cells in hematological and solid tumors. **(A)** is the incidence of serious adverse events of hematological tumors; **(B)** is the incidence of serious adverse events in solid tumors.

In this study, nervous system SAEs occurred in 2 cases (2.17%) during the treatment of solid tumors. Confusion (1 case) and encephalopathy (1 case) were the SAEs (**Table 4**). There were 3 cases (3.26%) of SAEs in Immune system and the main SAEs was CRS (**Table 4**). The type of SAEs of CAR-T cells in the treatment of solid tumors is basically similar to that of the hematological tumors. However, no cardiovascular adverse events were found in the included studies. In addition, this study have found that the incidence of neurological SAEs and CRS in solid tumors is lower than that in hematological tumors (**Figure 2**). Similarly, a clinical study (NCT03874897) conducted by C. Qi et al. (64) evaluated the safety and efficacy of CAR-T cells targeting CLDN18.2 in the treatment of gastric cancer. Results of 37 patients treated, 94.6% had grade 1 or 2 CRS. However, no deaths have been reported. Besides, Y. Liu et al. (65) conducted a phase I trial (NCT01869166) to evaluate the safety and efficacy of autologous anti-EGFR CAR-T cells in patients with metastatic prostate cancer in 14 patients. No SAEs such as cardiovascular system, nervous system, blood system and CRS were found. Furthermore, Y. Zhang et al. (66) also evaluated the safety of EGFR-targeted CAR-T cells in the treatment of small cell lung cancer. The most common adverse events were grade 1 to 3 fever. No patients had grade 4 adverse events or severe CRS. The tumor-killing sites of CAR-T cells are different in hematological tumors than in solid tumors. Solid tumors are more limited to tumor tissues due to targeted guidance, while hematological tumors cover the entire blood system due to tumor cells dispersed in the blood system. Therefore, some SAEs of CAR-T cells in hematological tumors may be more severe than those in solid tumors.

In this study, Respiratory, thoracic and mediastinal SAEs, Infection-related SAEs, Blood and lymphatic system SAEs, General SAEs occurred in 13 cases (14.13%), 5 cases (5.43%), 8 cases (8.70%) and 33 cases (35.87%) respectively (**Table 4**). Similarly, Z. Zhao et al. (55) conducted a meta-analysis involving 10 studies (94 patients) that reported the occurrence of adverse events during the treatment of digestive system tumors with CAR-T cells. The study found that the five most common side effects were fever, lymphadenia, pain other than abdominal pain, thrombocytopenia and fatigue. The specific SAEs types were basically the same as those of hematological tumors. Interestingly, these findings suggest that CAR-T cells SAEs in solid tumors and hematological tumors are similar.

## 3 The pathological mechanism of SAEs in the treatment of malignant tumors by CAR-T cells

It has been established that CRS and ICANS are the two major causes of all complications associated with CAR-T cells therapy (31, 42, 67, 68). In light of this, understanding the pathological mechanism of CRS and ICANS is of theoretical importance when dealing with patients with severe complications.

CRS is a systemic inflammatory response, and current studies have shown that it can be induced by a variety of factors, including severe infection, followed by drugs, such as CAR-T cells and monoclonal antibodies (69–74). Severe viral infections such as influenza and COVID-19 can also trigger CRS through massive immune and non-immune cell stimulation (75). CRS is usually associated with tumor load and usually occurs between day 1 and week 2 after CAR-T cells infusion (76, 77). All systems of the body are affected by CRS, including fever, myalgia, anorexia, hypotension, tachycardia, arrhythmia, shortness of breath and hypoxia, coagulopathy, respiratory failure, shock and organ dysfunction etc (42, 46, 48, 57, 78).

Upon interaction of CAR-T cells with the corresponding target antigen, inflammatory cytokines and chemokines such as interferon (IFN) γ, tumor necrosis factor (TNF)α, granulocyte macrophage colony-stimulating factor (GM-CSF), interleukin (IL)-6, IL-10 are released (79–82). High secretion of these cytokines can lead to systemic inflammatory response-CRS. However, not all of these cytokines were secreted by activated CAR-T cells. Activating peripheral immune and non-immune cells such as monocytes, macrophages, dendritic cells, and endothelial cells is accomplished by CAR-T cells binding to antigens on tumor cells (83, 84). It has been shown that xenogeneic models emphasize the role of host immune cells in CRS pathogenesis, suggesting that IL-6 is primarily released by monocytes, macrophages, and dendritic cells, not CAR-T cells (82, 85, 86). Since IL-6 plays a key role in CRS, depleting macrophages (87) and eliminating monocytes (86) may reduce its severity. Further, inhibiting GM-CSF signaling alleviates symptoms of CRS (88, 89).

ICANS was another cause of SAEs during CAR-T cells therapy (40, 76, 90–92). In addition to CD19, CAR- T cells targeting CD22, BCMA, and other hematopoietic antigens have also been observed for neurotoxicity (11, 13, 93–95). Other treatments involving immune effector cells have also been reported to cause similar neurotoxic effects (96, 97). Therefore, the neurotoxicity of CAR-T cells was renamed ICANS (80, 98).ICANS can occur in conjunction with or independently of CRS (83, 99, 100). ICANS occurs independently and the general neurological symptoms tend to be mild (35). Typically, ICANS appear 4-5 days after CAR-T cells therapy, but delayed ICANS have also been reported after CAR-T cells therapy (26, 34, 98).

ICANS typically manifest as disturbances in attention and consciousness, and expressive aphasia is considered a fairly specific early sign of ICANS (26). ICANS can further develop into low levels of consciousness, coma, epilepsy, motor weakness, and cerebral edema. All cases of fatal cerebral edema are associated with CRS (34, 35), and severe CRS has been shown to be associated with severe ICANS (92, 101, 102). At present, relatively little is known about the pathophysiology of ICANS. ICANS have been associated with CAR-T cells transport in the central nervous system (98, 103, 104), passive diffusion of cytokines into the central nervous system (26, 34, 105), endothelial activation with impaired blood-brain barrier (26, 34), activation of microglia and myeloid cells in the central nervous system with secretion of IL-1 and IL-6 (85, 86).

## 4 Strategies to deal with SAEs of CAR-T cells therapy

The primary cause of CAR-T cells-associated SAEs is CRS and ICANS (31, 42, 67, 68), so treating SAEs involves preventing CRS and ICANS, as well as alleviating symptoms (67, 106). The specific measures were on one hand to optimize the CAR-T cells structure to reduce cytokine release. On the other hand, clinical management should be strengthened to find and correct CRS and ICANS in time to reduce the occurrence of related SAEs.

### 4.1 Optimization of CAR-T cells structure

Stable proliferation and activation of CAR-T cells in the tumor microenvironment are the prerequisite for tumor killing, but safety is also crucial (107). Endogenous non-effector immune cells are also expanded during CAR-T cells therapy. In studies on CRS, monocytes and macrophages were found to be the major source of cytokines associated with severe manifestations (31, 108). A large number of preclinical studies have demonstrated that different CAR-T cells structures and scFv sequences can produce different tumor killing efficacy (17, 109–112). Additionally, CAR-T cells must be positively regulated by a large number of cytokines in order to kill tumors. Therefore, CAR-T cells constructs were designed to activate and maintain CAR-T cells while attenuating monocyte and macrophage activation. The structure of CAR-T cells is correlated with the incidence of CRS. To reduce the risk of CRS, newly designed next-generation CAR-T cells therapy is being developed for hematopoietic malignancies and solid tumors. S. Balagopal et al (113) have discussed Six interesting approaches to control cytokine production in CAR-T cells therapy: adaptor-based strategies, orthogonal cytokine–receptor pairs, regulation of macrophage cytokine activity, autonomous neutralization of key cytokines, kill switches and methods of reversible suppression of CARs. With these strategies, future CAR-T cells therapies will be designed to preemptively inhibit CRS, minimizing patient suffering and maximizing the number of patients who benefit.

Furthermore, the selection of different costimulatory domains by CAR-T cells affected the occurrence of ICANS. Approximately 45% of patients treated with CAR-T cells containing CD28 as a costimulatory domain develop high-grade ICANS (39, 91, 92, 114, 115). However, ICANS was less common during treatment with CAR-T cells using 4-1 BB as the co-stimulatory domain, with 13% of patients experiencing severe ICANS (76, 77). W. Luo et al. (116)conducted a meta-analysis involving 52 studies including 2,004 patients. Hematotoxicity analysis of CD19 CAR-T cells subsets demonstrated that 4-1BB, as a costimulatory domain, had less hematotoxicity than CD28. Therefore, it is of great significance to optimize the selection of co-stimulatory domain to avoid the occurrence of ICANS.

The development of relatively specific targets for solid tumors is also crucial. It is well known that specific targets have not been found in the treatment of solid tumors, and only tumor-associated targets are used in CAR-T cells (117, 118). This leads to the possibility that CAR-T cells targeting such targets may cause cytotoxicity outside the tumor. R. A. Morgan et al. (119) reported that CAR-T cells targeting HER-2 in the treatment of colorectal cancer, because CAR-T cells simultaneously targeted and killed the patient’s pleural cells, the patient eventually died of respiratory failure. The above case report indicates that it is crucial to select relatively specific targets in the treatment of solid tumors with CAR-T cells. Therefore, the treatment of solid tumors with CAR-T cells should first optimize the selection of targets, and then design more optimal CAR frames to reduce the occurrence of CRS while killing tumors.

### 4.2 Clinical management and medication

The management of SAEs in CAR-T cells therapy is actually primarily about controlling CRS. Standardized grading of clinical adverse events was first required using the common terminology criteria for adverse events (CTCAE) (120) and CAR-T cells therapy-related toxicity (CARTOX) scoring systems. If CRS is suspected, the patient should be graded at least twice a day as the patient’s condition changes (121). Management of CRS should be determined on a hierarchical basis, and low-grade CRS can be managed mainly through supportive care. The anti-IL-6 receptor antagonist tocilizumab and/or corticosteroids are considered when high-grade CRS and persistent refractory fever or fluid-refractory hypotension occur together (98).

The use of steroids for the suppression of excessive inflammatory responses and CRS has been proven in clinical experience (67). Several views exist regarding when and how corticosteroids should be administered. Some choose to use corticosteroids as a first-line agent, while others don’t (83). It is important to recognize that corticosteroids have general effects on the immune system, which may also affect the antitumor efficacy and the amplification and persistence of CAR-T cells *in vivo (* 122). Therefore, steroids should be avoided as first-line treatment, but used when ablating CAR-T cells is necessary in patients with severe CRS and who are resistant to other treatments. Furthermore, steroids are recommended for patients who are experiencing adverse neurological effects.

Tocilizumab is a humanized monoclonal antibody to the IL-6 receptor that inhibits the IL-6 signaling pathway (76, 123). It was approved by the FDA in 2017 as the first treatment for CRS-related toxicity following CAR-T cells infusion. Tocilizumab controlled CRS but did not significantly reduce CAR-T cells activity. The favorable effect of a single injection in patients with CRS induced by CAR-T cells therapy strongly suggests that IL-6 blocking may constitute a novel therapeutic approach for the treatment of severe systemic inflammatory responses. In patients who respond, fever and low blood pressure improve within a few hours, while in some patients supportive treatment is needed for several days. H. Liu et al. (124) evaluated the antitumor effect and safety of PD-L1-targeted CAR-T cells in patients with non-small cell lung cancer through a phase I clinical study. One patient in the trial developed severe CRS with symptoms of pneumonia and respiratory failure. The patient was given oxygen and treated with intravenous tocilizumab and methylprednisolone. The patient’s symptoms improved quickly and the lung inflammation gradually subsided. Besides, K. Qi et al. (125) analyzed the adverse events after treatment in 126 patients with hematologic malignancies who received CAR-T cells therapy. The results showed that cardiac adverse events associated with CAR-T cells therapy were common and related to the development of CRS. For patients with grade 3-5 CRS, timely administration of corticosteroids and/or tocilizumab can effectively prevent the occurrence and development of cardiac disease. However, a large number of patients are resistant to tocilizumab (98). Another therapeutic agent is a monoclonal antibody targeting IL-6, siltuximab, which has a higher affinity for IL-6 than tocilizumab for the IL6 receptor, making it a potential smoke screen for CRS treatment (126). Siltuximab is encouraged in patients who do not respond to tocilizumab and corticosteroids.

Clinically, because the clinical manifestations of infection and CRS are very similar (28, 127). Thus, diagnosis of infection becomes difficult when CRS are present. However, the treatment of CRS and infection is different (83, 98). CRS can be successfully improved with IL-6 receptor inhibitors and corticosteroids, whereas infection requires immediate initiation of antibiotic therapy (83). Therefore, it is necessary to distinguish between infections and CRS for appropriate treatment in CAR-T cells therapy. H. Luo et al. (49) selected 109 cases from three clinical trials (ChiCTR-OPN-16008526, ChiCTR-OPC-16009113, ChiCTR-OPN-16009847) to analyze the characteristics of infection events within 30 days after CAR-T cells infusion. The “IL-6 double peak” was found in most patients with life-threatening infections. Secondly, the prediction model constructed by IL-8, IL-1β and IFN-γ has high sensitivity and specificity for predicting life-threatening infections. This study indicates that the selection of effective markers during CAR-T cells therapy is very important for the diagnosis of life-threatening infections during CAR-T cells therapy and helps to reduce the risk of infection-induced death.

In addition, the classification and management of ICANS is also particularly important. It is recommended to have a neurological assessment prior to starting CAR-T cells therapy and to have one every day for the first 10 days following the infusion of CAR-T cells (128). Most commonly used tools for detecting and monitoring ICANS are the ICE score and ICANS grading system. The management of patients with grade 3 or greater ICANS should be conducted in the ICU, including the provision of airway support if the patient is not conscious (38, 128).

Corticosteroids are the mainstay of treatment for ICANS. While corticosteroids may reduce the antitumor effects of CD19 CAR-T cells (122, 129), they are appropriate for the treatment of moderate to severe ICANS due to their ICANS reversal effect. Generally, patients with low initial consciousness level are recommended to use dexamethasone for 1-3 days. The treatment for grade 4 ICANS includes 1000 mg of methylprednisolone, as the patient may not be able to wake up, may be epileptic, or may exhibit imaging characteristics of cerebral edema (128, 130). For patients with severe ICANS characterized by cerebral edema, some groups advocate supportive measures to manage elevated intracranial pressure, including the use of intracranial pressure monitors, decreasing intracranial pressure, etc (38, 128).

Tocilizumab can be used to treat ICANS, with the greatest benefit when ICANS occurs early and/or in conjunction with CRS (38, 98). It may be due to the increased permeability of the blood-brain barrier in the early stages, which facilitates tocilizumab ‘s entry into the brain (98). Studies have shown that tocilizumab may aggravate neurotoxicity, and the proposed mechanism is that blocking IL-6 receptors with tocilizumab may lead to increased circulating IL-6 in the central nervous system. Therefore, treatment with a monoclonal antibody (siltuximab) directly binding to IL-6 is recommended (38, 131, 132). Siltuximab directly bound to IL-6 may be more beneficial in isolated ICANS cases (38). Preclinical studies suggest that future therapies such as monoclonal antibodies targeting IL-1 may benefit ICANS, although clinical evidence is unproven for the time being (86, 130, 133). In early trials, when ICANS appeared, antiepileptic drugs were prophylactically administered to the clinic. The benefits of prophylactic use of antiepileptic drugs, which have not been proven to reduce epilepsy complications definitively, remain controversial (26, 38, 105). The use of benzodiazepines to treat sudden seizures is effective in most cases, although refractory or prolonged seizures may also occur (26, 105). Levetiracetam appears to be the preferred antiepileptic agent for ICANS patients, possibly because of its low incidence of drug interactions and good safety (38, 98).

Based on available evidence and clinical experience, the NCCN Guidelines for management of immunotherapy-related complications also provided recommendations on monitoring patients receiving CAR-T cells therapy (22). Patients with underlying organ dysfunction may have additional adverse events when receiving CAR-T cells therapy, and multidisciplinary intervention is particularly important for these patients when SAEs occur. Since SAEs caused by CAR-T cells can be seen in various organs of the body, the importance of multidisciplinary collaboration in CAR-T cells therapy is emphasized finally.

## 5 Discussion

CAR-T cells technology is a major breakthrough in the field of cancer, as the star of tumor immunotherapy has brought light to patients with advanced tumors, especially B cell-derived hematological tumors and multiple myeloma (134–136). More and more studies have shown its efficacy in a variety of cancers, and a large number of clinical studies on hematological tumors and solid tumors are ongoing. However, data from a growing number of clinical trials indicate that all CAR-T cells therapies have unique adverse events, such as CRS and ICANS (67, 137). Its adverse events can cause clinical symptoms in many systems of the whole body, manifested as a high incidence, serious can endanger life (68, 138). Therefore, it is important to pay attention to the occurrence of SAEs during CAR-T cells therapy for advancing the treatment of advanced malignant tumors.

In this review, we summarize a subset of studies in the treatment of hematological malignancies and solid tumors and analyze the occurrence of clinical SAEs in the included studies. In combination with published clinical studies, CRS was found to be associated with SAEs in all major systemic systems. In addition, all cases of severe ICANS were found to be associated with CRS (34, 35).Thus, we found that CRS may be a major cause of life-threatening adverse events in the treatment of malignant tumors with CAR-T cells. In fact, cytokines play a dual role in CAR-T cells therapy. On the one hand, they activate CAR-T cells to kill tumor cells (110, 111, 139, 140). At the same time, it activates the non-effector immune cells and then produces a large number of negative cytokines, which leads to the damage of the body (81, 85, 141). Therefore, to be widely used in the treatment of malignant tumors in the future, CAR-T cells technology must be further optimized in the design process to activate CAR-T cells while reducing the impact on non-effector immune cells.

This review also provides an overview of the management and treatment of SAEs during CAR-T cells therapy. In view of the high incidence of SAEs in the clinical application of CAR-T cells (67, 142), it is necessary to closely monitor the vital signs of patients in clinical application, timely evaluate the CRS grade, and timely give standardized treatment according to the grade (67, 138). Most SAEs can be reversed (137), and patients will benefit most from timely multidisciplinary consultation.

In addition, the comparison of SAEs after CAR-T cells therapy for hematological and solid tumors included in this review may be different. Firstly, cardiac SAEs were not found in the solid tumor study. Secondly, the incidence of SAEs of nervous system and CRS in solid tumors is lower than that in hematological tumors (**Figure 2**). W. Lei et al. (143) included a total of 2592 patients in 84 studies for meta-analysis, and analyzed the differences in the incidence of CRS and ICANS of CAR-T cells in different tumor types. The results showed that the incidence of CRS and ICANS in hematologic malignancies was significantly higher than that in solid tumors. Our findings are confirmed by this study. CAR-T cells mainly exist in tumor tissues during the treatment of solid tumors because of the targeted guidance. Nevertheless, CAR-T cells need to be disseminated throughout the blood system in the treatment of hematological tumors, so the cytokines produced may be more readily disseminated in the body, which may be the reason for the difference in the incidence and severity of some adverse events during the treatment of hematologic and solid tumors with CAR-T cells therapy.

## 6 Conclusion

In conclusion, CAR-T cells technology can produce a variety of SAEs in the treatment of malignant tumors, which can occur in various systems of the body and can be life-threatening in severe cases. Studies have shown that CRS and ICANS may be the main causes of the above clinically SAEs. Therefore, through strict clinical grading and management of CRS and ICANS, most of the adverse events can be alleviated.

## Author contributions

All authors conceptualized and wrote the manuscript.XC and XK additionally performed literature and data analysis. All authors contributed to the article and approved the submitted version.

## References

[1] Huo JL Fu WJ Liu ZH Lu N Jia XQ Liu ZS . Research advance of natural products in tumor immunotherapy. Front Immunol (2022) 13:972345. doi: 10.3389/fimmu.2022.972345 36159787 PMC9494295

[2] Lesch S Gill S . The promise and perils of immunotherapy. Blood Adv (2021) 5(18):3709–25. doi: 10.1182/bloodadvances.2021004453C PMC8945582 34581774

[3] June CH Sadelain M . Chimeric antigen receptor therapy. N Engl J Med (2018) 379(1):64–73. doi: 10.1056/NEJMra1706169 29972754 PMC7433347

[4] Feins S Kong W Williams EF Milone MC Fraietta JA . An introduction to chimeric antigen receptor (CAR) T-cell immunotherapy for human cancer. Am J Hematol (2019) 94(S1):S3–s9. doi: 10.1002/ajh.25418 30680780

[5] Miao L Zhang Z Ren Z Li Y . Reactions related to CAR-T cell therapy. Front Immunol (2021) 12:663201. doi: 10.3389/fimmu.2021.663201 33995389 PMC8113953

[6] Wang J Mou N Yang Z Li Q Jiang Y Meng J . Efficacy and safety of humanized anti-CD19-CAR-T therapy following intensive lymphodepleting chemotherapy for refractory/relapsed b acute lymphoblastic leukaemia. Br J Haematol (2020) 191(2):212–22. doi: 10.1111/bjh.16623 PMC7687133 32232846

[7] Geyer MB Rivière I Sénéchal B Wang X Wang Y Purdon TJ . Autologous CD19-targeted CAR T cells in patients with residual CLL following initial purine analog-based therapy. Mol Ther (2018) 26(8):1896–905. doi: 10.1016/j.ymthe.2018.05.018 PMC6094824 29910179

[8] Abramson JS . Anti-CD19 CAR T-cell therapy for b-cell non-Hodgkin lymphoma. Transfus Med Rev (2020) 34(1):29–33. doi: 10.1016/j.tmrv.2019.08.003 31677848

[9] Gauthier J Bezerra ED Hirayama AV Fiorenza S Sheih A Chou CK . Factors associated with outcomes after a second CD19-targeted CAR T-cell infusion for refractory b-cell malignancies. Blood (2021) 137(3):323–35. doi: 10.1182/blood.2020006770 PMC7819764 32967009

[10] Zhang J Hu Y Yang J Li W Zhang M Wang Q . Non-viral, specifically targeted CAR-T cells achieve high safety and efficacy in b-NHL. Nature (2022) 609(7926):369–74. doi: 10.1038/s41586-022-05140-y PMC9452296 36045296

[11] Fry TJ Shah NN Orentas RJ Stetler-Stevenson M Yuan CM Ramakrishna S . CD22-targeted CAR T cells induce remission in b-ALL that is naive or resistant to CD19-targeted CAR immunotherapy. Nat Med (2018) 24(1):20–8. doi: 10.1038/nm.4441 PMC5774642 29155426

[12] Shalabi H Wolters PL Martin S Toledo-Tamula MA Roderick MC Struemph K . Systematic evaluation of neurotoxicity in children and young adults undergoing CD22 chimeric antigen receptor T-cell therapy. J Immunother (2018) 41(7):350–8. doi: 10.1097/cji.0000000000000241 PMC6086728 30048343

[13] Zhao WH Liu J Wang BY Chen YX Cao XM Yang Y . A phase 1, open-label study of LCAR-B38M, a chimeric antigen receptor T cell therapy directed against b cell maturation antigen, in patients with relapsed or refractory multiple myeloma. J Hematol Oncol (2018) 11(1):141. doi: 10.1186/s13045-018-0681-6 30572922 PMC6302465

[14] Raje N Berdeja J Lin Y Siegel D Jagannath S Madduri D . Anti-BCMA CAR T-cell therapy bb2121 in relapsed or refractory multiple myeloma. N Engl J Med (2019) 380(18):1726–37. doi: 10.1056/NEJMoa1817226 PMC8202968 31042825

[15] Cohen AD Garfall AL Stadtmauer EA Melenhorst JJ Lacey SF Lancaster E . B cell maturation antigen-specific CAR T cells are clinically active in multiple myeloma. J Clin Invest (2019) 129(6):2210–21. doi: 10.1172/jci126397 PMC6546468 30896447

[16] Staudt RE Carlson RD Snook AE . Targeting gastrointestinal cancers with chimeric antigen receptor (CAR)-T cell therapy. Cancer Biol Ther (2022) 23(1):127–33. doi: 10.1080/15384047.2022.2033057 PMC8820794 35129050

[17] Zhao R Cui Y Zheng Y Li S Lv J Wu Q . Human hyaluronidase PH20 potentiates the antitumor activities of mesothelin-specific CAR-T cells against gastric cancer. Front Immunol (2021) 12:660488. doi: 10.3389/fimmu.2021.660488 34326835 PMC8313856

[18] Pang N Shi J Qin L Chen A Tang Y Yang H . IL-7 and CCL19-secreting CAR-T cell therapy for tumors with positive glypican-3 or mesothelin. J Hematol Oncol (2021) 14(1):118. doi: 10.1186/s13045-021-01128-9 34325726 PMC8323212

[19] Beatty GL O’Hara MH Lacey SF Torigian DA Nazimuddin F Chen F . Activity of mesothelin-specific chimeric antigen receptor T cells against pancreatic carcinoma metastases in a phase 1 trial. Gastroenterology (2018) 155(1):29–32. doi: 10.1053/j.gastro.2018.03.029 29567081 PMC6035088

[20] You F Jiang L Zhang B Lu Q Zhou Q Liao X . Phase 1 clinical trial demonstrated that MUC1 positive metastatic seminal vesicle cancer can be effectively eradicated by modified anti-MUC1 chimeric antigen receptor transduced T cells. Sci China Life Sci (2016) 59(4):386–97. doi: 10.1007/s11427-016-5024-7 26961900

[21] Zhou JT Liu JH Song TT Ma B Amidula N Bai C . EGLIF-CAR-T cells secreting PD-1 blocking antibodies significantly mediate the elimination of gastric cancer. Cancer Manag Res (2020) 12:8893–902. doi: 10.2147/cmar.S260915 PMC7520465 33061585

[22] Thompson JA Schneider BJ Brahmer J Achufusi A Armand P Berkenstock MK . Management of immunotherapy-related toxicities, version 1.2022, NCCN clinical practice guidelines in oncology. J Natl Compr Canc Netw (2022) 20(4):387–405. doi: 10.6004/jnccn.2022.0020 35390769

[23] Greenbaum U Kebriaei P Srour SA Olson A Bashir Q Neelapu SS . Chimeric antigen receptor T-cell therapy toxicities. Br J Clin Pharmacol (2021) 87(6):2414–24. doi: 10.1111/bcp.14403 32463929

[24] Turtle CJ Hanafi LA Berger C Hudecek M Pender B Robinson E . Immunotherapy of non-hodgkin’s lymphoma with a defined ratio of CD8+ and CD4+ CD19-specific chimeric antigen receptor-modified T cells. Sci Transl Med (2016) 8(355):355ra116. doi: 10.1126/scitranslmed.aaf8621 PMC5045301 27605551

[25] Hay KA Hanafi LA Li D Gust J Liles WC Wurfel MM . Kinetics and biomarkers of severe cytokine release syndrome after CD19 chimeric antigen receptor-modified T-cell therapy. Blood (2017) 130(21):2295–306. doi: 10.1182/blood-2017-06-793141 PMC5701525 28924019

[26] Santomasso BD Park JH Salloum D Riviere I Flynn J Mead E . Clinical and biological correlates of neurotoxicity associated with CAR T-cell therapy in patients with b-cell acute lymphoblastic leukemia. Cancer Discovery (2018) 8(8):958–71. doi: 10.1158/2159-8290.Cd-17-1319 PMC6385599 29880584

[27] Lundh S Maji S Melenhorst JJ . Next-generation CAR T cells to overcome current drawbacks. Int J Hematol (2021) 114(5):532–43. doi: 10.1007/s12185-020-02923-9 32594314

[28] Xu XJ Tang YM . Cytokine release syndrome in cancer immunotherapy with chimeric antigen receptor engineered T cells. Cancer Lett (2014) 343(2):172–8. doi: 10.1016/j.canlet.2013.10.004 24141191

[29] Wei Z Cheng Q Xu N Zhao C Xu J Kang L . Investigation of CRS-associated cytokines in CAR-T therapy with meta-GNN and pathway crosstalk. BMC Bioinf (2022) 23(1):373. doi: 10.1186/s12859-022-04917-2 PMC9469618 36100873

[30] Maus MV Alexander S Bishop MR Brudno JN Callahan C Davila ML . Society for immunotherapy of cancer (SITC) clinical practice guideline on immune effector cell-related adverse events. J Immunother Cancer (2020) 8(2):e001511. doi: 10.1136/jitc-2020-001511 33335028 PMC7745688

[31] Cosenza M Sacchi S Pozzi S . Cytokine release syndrome associated with T-Cell-Based therapies for hematological malignancies: Pathophysiology, clinical presentation, and treatment. Int J Mol Sci (2021) 22(14):7652. doi: 10.3390/ijms22147652 34299273 PMC8305850

[32] Zhang Z Miao L Ren Z Tang F Li Y . Gene-edited interleukin CAR-T cells therapy in the treatment of malignancies: Present and future. Front Immunol (2021) 12:718686. doi: 10.3389/fimmu.2021.718686 34386015 PMC8353254

[33] Ying Z Song Y Zhu J . Effectiveness and safety of anti-CD19 chimeric antigen receptor-T cell immunotherapy in patients with Relapsed/Refractory Large b-cell lymphoma: A systematic review and meta-analysis. Front Pharmacol (2022) 13:834113. doi: 10.3389/fphar.2022.834113 35548364 PMC9081610

[34] Gust J Hay KA Hanafi LA Li D Myerson D Gonzalez-Cuyar LF . Endothelial activation and blood-brain barrier disruption in neurotoxicity after adoptive immunotherapy with CD19 CAR-T cells. Cancer Discovery (2017) 7(12):1404–19. doi: 10.1158/2159-8290.Cd-17-0698 PMC5718945 29025771

[35] Gust J Ceppi F Turtle CJ . Chapter 7 - neurotoxicities after CAR T-cell immunotherapy. In: Lee DW Shah NN , editors. Chimeric antigen receptor T-cell therapies for cancer. Elsevier (2020) 83–105. doi: 10.1016/B978-0-323-66181-2.00007-X

[36] Shao M Yu Q Teng X Guo X Wei G Xu H . CRS-related coagulopathy in BCMA targeted CAR-T therapy: a retrospective analysis in a phase I/II clinical trial. Bone Marrow Transplant (2021) 56(7):1642–50. doi: 10.1038/s41409-021-01226-9 33608658

[37] Zhou J Zhang Y Shan M Zong X Geng H Li J . Cytopenia after chimeric antigen receptor T cell immunotherapy in relapsed or refractory lymphoma. Front Immunol (2022) 13:997589. doi: 10.3389/fimmu.2022.997589 36131934 PMC9484486

[38] Gust J Taraseviciute A Turtle CJ . Neurotoxicity associated with CD19-targeted CAR-T cell therapies. CNS Drugs (2018) 32(12):1091–101. doi: 10.1007/s40263-018-0582-9 PMC7295115 30387077

[39] Locke FL Ghobadi A Jacobson CA Miklos DB Lekakis LJ Oluwole OO . Long-term safety and activity of axicabtagene ciloleucel in refractory large b-cell lymphoma (ZUMA-1): a single-arm, multicentre, phase 1-2 trial. Lancet Oncol (2019) 20(1):31–42. doi: 10.1016/s1470-2045(18)30864-7 30518502 PMC6733402

[40] Schuster SJ Bishop MR Tam CS Waller EK Borchmann P McGuirk JP . Tisagenlecleucel in adult relapsed or refractory diffuse Large b-cell lymphoma. N Engl J Med (2019) 380(1):45–56. doi: 10.1056/NEJMoa1804980 30501490

[41] Lv L Wu Y Shi H Sun X Deng Z Huo H . Efficacy and safety of chimeric antigen receptor T-cells treatment in central nervous system lymphoma: a PRISMA-compliant single-arm meta-analysis. Cancer Immunol Immunother (2022). doi: 10.1007/s00262-022-03246-w PMC10991213 35796863

[42] Gajra A Zettler ME Phillips EG Jr. Klink AJ Jonathan KK Fortier S . Neurological adverse events following CAR T-cell therapy: a real-world analysis. Immunotherapy (2020) 12(14):1077–82. doi: 10.2217/imt-2020-0161 32808566

[43] Karschnia P Jordan JT Forst DA Arrillaga-Romany IC Batchelor TT Baehring JM . Clinical presentation, management, and biomarkers of neurotoxicity after adoptive immunotherapy with CAR T cells. Blood (2019) 133(20):2212–21. doi: 10.1182/blood-2018-12-893396 30808634

[44] Pan J Tan Y Wang G Deng B Ling Z Song W . Donor-derived CD7 chimeric antigen receptor T cells for T-cell acute lymphoblastic leukemia: First-in-Human, phase I trial. J Clin Oncol (2021) 39(30):3340–51. doi: 10.1200/jco.21.00389 34324392

[45] Benjamin R Graham C Yallop D Jozwik A Mirci-Danicar OC Lucchini G . Genome-edited, donor-derived allogeneic anti-CD19 chimeric antigen receptor T cells in paediatric and adult b-cell acute lymphoblastic leukaemia: results of two phase 1 studies. Lancet (2020) 396(10266):1885–94. doi: 10.1016/s0140-6736(20)32334-5 PMC11773457 33308471

[46] Rejeski K Kunz WG Rudelius M Bücklein V Blumenberg V Schmidt C . Severe candida glabrata pancolitis and fatal aspergillus fumigatus pulmonary infection in the setting of bone marrow aplasia after CD19-directed CAR T-cell therapy - a case report. BMC Infect Dis (2021) 21(1):121. doi: 10.1186/s12879-020-05755-4 33509115 PMC7841988

[47] Goldman A Maor E Bomze D Liu JE Herrmann J Fein J . Adverse cardiovascular and pulmonary events associated with chimeric antigen receptor T-cell therapy. J Am Coll Cardiol (2021) 78(18):1800–13. doi: 10.1016/j.jacc.2021.08.044 PMC8562317 34711339

[48] Totzeck M Michel L Lin Y Herrmann J Rassaf T . Cardiotoxicity from chimeric antigen receptor-T cell therapy for advanced malignancies. Eur Heart J (2022) 43(20):1928–40. doi: 10.1093/eurheartj/ehac106 PMC9123242 35257157

[49] Luo H Wang N Huang L Zhou X Jin J Li C . Inflammatory signatures for quick diagnosis of life-threatening infection during the CAR T-cell therapy. J Immunother Cancer (2019) 7(1):271. doi: 10.1186/s40425-019-0767-x 31640816 PMC6806557

[50] Gill J . Cardiovascular toxicities with chimeric antigen receptor T-cell therapy. Curr Cardiol Rev (2022) 19(1):2023–11. doi: 10.2174/1573403x18666220623152350 PMC10201875 35747980

[51] Steiner RE Banchs J Koutroumpakis E Becnel M Gutierrez C Strati P . Cardiovascular events in patients treated with chimeric antigen receptor T-cell therapy for aggressive b-cell lymphoma. Haematologica (2022) 107(7):1555–66. doi: 10.3324/haematol.2021.280009 PMC9244830 34758610

[52] Hanna KS Kaur H Alazzeh MS Thandavaram A Channar A Purohit A . Cardiotoxicity associated with chimeric antigen receptor (CAR)-T cell therapy for hematologic malignancies: A systematic review. Cureus (2022) 14(8):e28162. doi: 10.7759/cureus.28162 36148204 PMC9482759

[53] Alvi RM Frigault MJ Fradley MG Jain MD Mahmood SS Awadalla M . Cardiovascular events among adults treated with chimeric antigen receptor T-cells (CAR-T). J Am Coll Cardiol (2019) 74(25):3099–108. doi: 10.1016/j.jacc.2019.10.038 PMC6938409 31856966

[54] Guha A Addison D Jain P Gutierrez JM Ghosh A Roddie C . Cardiovascular events associated with chimeric antigen receptor T cell therapy: Cross-sectional FDA adverse events reporting system analysis. Biol Blood Marrow Transplant (2020) 26(12):2211–6. doi: 10.1016/j.bbmt.2020.08.036 32966880

[55] Zhao Z Zhang J Bian J Lu X . The efficacy and safety of chimeric antigen receptor T cells in digestive system cancers: a systematic review and meta-analysis. Ann Transl Med (2022) 10(9):508. doi: 10.21037/atm-21-5019 35928759 PMC9347051

[56] Telli Dizman G Aguado JM Fernández-Ruiz M . Risk of infection in patients with hematological malignancies receiving CAR T-cell therapy: systematic review and meta-analysis. Expert Rev Anti Infect Ther (2022) 20(11):1455–76. doi: 10.1080/14787210.2022.2128762 36148506

[57] Fishman JA Hogan JI Maus MV . Inflammatory and infectious syndromes associated with cancer immunotherapies. Clin Infect Dis (2019) 69(6):909–20. doi: 10.1093/cid/ciy1025 30520987

[58] Hill JA Li D Hay KA Green ML Cherian S Chen X . Infectious complications of CD19-targeted chimeric antigen receptor-modified T-cell immunotherapy. Blood (2018) 131(1):121–30. doi: 10.1182/blood-2017-07-793760 PMC5755046 29038338

[59] Park JH Romero FA Taur Y Sadelain M Brentjens RJ Hohl TM . Cytokine release syndrome grade as a predictive marker for infections in patients with relapsed or refractory b-cell acute lymphoblastic leukemia treated with chimeric antigen receptor T cells. Clin Infect Dis (2018) 67(4):533–40. doi: 10.1093/cid/ciy152 PMC6070095 29481659

[60] Maude SL Barrett D Teachey DT Grupp SA . Managing cytokine release syndrome associated with novel T cell-engaging therapies. Cancer J (2014) 20(2):119–22. doi: 10.1097/ppo.0000000000000035 PMC4119809 24667956

[61] Locke FL Neelapu SS Bartlett NL Siddiqi T Chavez JC Hosing CM . Phase 1 results of ZUMA-1: A multicenter study of KTE-C19 anti-CD19 CAR T cell therapy in refractory aggressive lymphoma. Mol Ther (2017) 25(1):285–95. doi: 10.1016/j.ymthe.2016.10.020 PMC5363293 28129122

[62] Nahas GR Komanduri KV Pereira D Goodman M Jimenez AM Beitinjaneh A . Incidence and risk factors associated with a syndrome of persistent cytopenias after CAR-T cell therapy (PCTT). Leuk Lymphoma (2020) 61(4):940–3. doi: 10.1080/10428194.2019.1697814 31793821

[63] Wang L Hong R Zhou L Ni F Zhang M Zhao H . New-onset severe cytopenia after CAR-T cell therapy: Analysis of 76 patients with relapsed or refractory acute lymphoblastic leukemia. Front Oncol (2021) 11:702644. doi: 10.3389/fonc.2021.702644 34277448 PMC8278328

[64] Qi C Gong J Li J Liu D Qin Y Ge S . Claudin18.2-specific CAR T cells in gastrointestinal cancers: phase 1 trial interim results. Nat Med (2022) 28(6):1189–98. doi: 10.1038/s41591-022-01800-8 PMC9205778 35534566

[65] Liu Y Guo Y Wu Z Feng K Tong C Wang Y . Anti-EGFR chimeric antigen receptor-modified T cells in metastatic pancreatic carcinoma: A phase I clinical trial. Cytotherapy (2020) 22(10):573–80. doi: 10.1016/j.jcyt.2020.04.088 32527643

[66] Zhang Y Zhang Z Ding Y Fang Y Wang P Chu W . Phase I clinical trial of EGFR-specific CAR-T cells generated by the piggyBac transposon system in advanced relapsed/refractory non-small cell lung cancer patients. J Cancer Res Clin Oncol (2021) 147(12):3725–34. doi: 10.1007/s00432-021-03613-7 PMC11801842 34032893

[67] Yáñez L Alarcón A Sánchez-Escamilla M Perales MA . How I treat adverse effects of CAR-T cell therapy. ESMO Open (2020) 4(Suppl 4):e000746. doi: 10.1136/esmoopen-2020-000746 32839196 PMC7451454

[68] Schubert ML Schmitt M Wang L Ramos CA Jordan K Müller-Tidow C . Side-effect management of chimeric antigen receptor (CAR) T-cell therapy. Ann Oncol (2021) 32(1):34–48. doi: 10.1016/j.annonc.2020.10.478 33098993

[69] Winkler U Jensen M Manzke O Schulz H Diehl V Engert A . Cytokine-release syndrome in patients with b-cell chronic lymphocytic leukemia and high lymphocyte counts after treatment with an anti-CD20 monoclonal antibody (rituximab, IDEC-C2B8). Blood (1999) 94(7):2217–24. doi: 10.1182/blood.V94.7.2217.419k02_2217_2224 10498591

[70] Freeman CL Morschhauser F Sehn L Dixon M Houghton R Lamy T . Cytokine release in patients with CLL treated with obinutuzumab and possible relationship with infusion-related reactions. Blood (2015) 126(24):2646–9. doi: 10.1182/blood-2015-09-670802 PMC4671111 26447188

[71] Wing MG Moreau T Greenwood J Smith RM Hale G Isaacs J . Mechanism of first-dose cytokine-release syndrome by CAMPATH 1-h: involvement of CD16 (FcgammaRIII) and CD11a/CD18 (LFA-1) on NK cells. J Clin Invest (1996) 98(12):2819–26. doi: 10.1172/jci119110 PMC507749 8981930

[72] Alig SK Dreyling M Seppi B Aulinger B Witkowski L Rieger CT . Severe cytokine release syndrome after the first dose of brentuximab vedotin in a patient with relapsed systemic anaplastic large cell lymphoma (sALCL): a case report and review of literature. Eur J Haematol (2015) 94(6):554–7. doi: 10.1111/ejh.12396 24913471

[73] de Vos S Forero-Torres A Ansell SM Kahl B Cheson BD Bartlett NL . A phase II study of dacetuzumab (SGN-40) in patients with relapsed diffuse large b-cell lymphoma (DLBCL) and correlative analyses of patient-specific factors. J Hematol Oncol (2014) 7:44. doi: 10.1186/1756-8722-7-44 24919462 PMC4065310

[74] Rotz SJ Leino D Szabo S Mangino JL Turpin BK Pressey JG . Severe cytokine release syndrome in a patient receiving PD-1-directed therapy. Pediatr Blood Cancer (2017) 64(12):e26642. doi: 10.1002/pbc.26642 28544595

[75] Moore JB June CH . Cytokine release syndrome in severe COVID-19. Science (2020) 368(6490):473–4. doi: 10.1126/science.abb8925 32303591

[76] Maude SL Laetsch TW Buechner J Rives S Boyer M Bittencourt H . Tisagenlecleucel in children and young adults with b-cell lymphoblastic leukemia. N Engl J Med (2018) 378(5):439–48. doi: 10.1056/NEJMoa1709866 PMC5996391 29385370

[77] Schuster SJ Svoboda J Chong EA Nasta SD Mato AR Anak Ö . Chimeric antigen receptor T cells in refractory b-cell lymphomas. N Engl J Med (2017) 377(26):2545–54. doi: 10.1056/NEJMoa1708566 PMC5788566 29226764

[78] Liu YH Zang XY Wang JC Huang SS Xu J Zhang P . Diagnosis and management of immune related adverse events (irAEs) in cancer immunotherapy. BioMed Pharmacother (2019) 120:109437. doi: 10.1016/j.biopha.2019.109437 31590992

[79] Wang Z Han W . Biomarkers of cytokine release syndrome and neurotoxicity related to CAR-T cell therapy. biomark Res (2018) 6:4. doi: 10.1186/s40364-018-0116-0 29387417 PMC5778792

[80] Lee DW Santomasso BD Locke FL Ghobadi A Turtle CJ Brudno JN . ASTCT consensus grading for cytokine release syndrome and neurologic toxicity associated with immune effector cells. Biol Blood Marrow Transplant (2019) 25(4):625–38. doi: 10.1016/j.bbmt.2018.12.758 PMC12180426 30592986

[81] Frey N Porter D . Cytokine release syndrome with chimeric antigen receptor T cell therapy. Biol Blood Marrow Transplant (2019) 25(4):e123–7. doi: 10.1016/j.bbmt.2018.12.756 30586620

[82] Titov A Petukhov A Staliarova A Motorin D Bulatov E Shuvalov O . The biological basis and clinical symptoms of CAR-T therapy-associated toxicites. Cell Death Dis (2018) 9(9):897. doi: 10.1038/s41419-018-0918-x 30181581 PMC6123453

[83] Lee DW Gardner R Porter DL Louis CU Ahmed N Jensen M . Current concepts in the diagnosis and management of cytokine release syndrome. Blood (2014) 124(2):188–95. doi: 10.1182/blood-2014-05-552729 PMC4093680 24876563

[84] Liu Q Zhou YH Yang ZQ . The cytokine storm of severe influenza and development of immunomodulatory therapy. Cell Mol Immunol (2016) 13(1):3–10. doi: 10.1038/cmi.2015.74 26189369 PMC4711683

[85] Giavridis T van der Stegen SJC Eyquem J Hamieh M Piersigilli A Sadelain M . CAR T cell-induced cytokine release syndrome is mediated by macrophages and abated by IL-1 blockade. Nat Med (2018) 24(6):731–8. doi: 10.1038/s41591-018-0041-7 PMC6410714 29808005

[86] Norelli M Camisa B Barbiera G Falcone L Purevdorj A Genua M . Monocyte-derived IL-1 and IL-6 are differentially required for cytokine-release syndrome and neurotoxicity due to CAR T cells. Nat Med (2018) 24(6):739–48. doi: 10.1038/s41591-018-0036-4 29808007

[87] van der Stegen SJ Davies DM Wilkie S Foster J Sosabowski JK Burnet J . Preclinical *in vivo* modeling of cytokine release syndrome induced by ErbB-retargeted human T cells: identifying a window of therapeutic opportunity? J Immunol (2013) 191(9):4589–98. doi: 10.4049/jimmunol.1301523 24062490

[88] Sachdeva M Duchateau P Depil S Poirot L Valton J . Granulocyte-macrophage colony-stimulating factor inactivation in CAR T-cells prevents monocyte-dependent release of key cytokine release syndrome mediators. J Biol Chem (2019) 294(14):5430–7. doi: 10.1074/jbc.AC119.007558 PMC6462525 30804212

[89] Sterner RM Sakemura R Cox MJ Yang N Khadka RH Forsman CL . GM-CSF inhibition reduces cytokine release syndrome and neuroinflammation but enhances CAR-T cell function in xenografts. Blood (2019) 133(7):697–709. doi: 10.1182/blood-2018-10-881722 30463995 PMC6376281

[90] Gardner RA Finney O Annesley C Brakke H Summers C Leger K . Intent-to-treat leukemia remission by CD19 CAR T cells of defined formulation and dose in children and young adults. Blood (2017) 129(25):3322–31. doi: 10.1182/blood-2017-02-769208 PMC5482103 28408462

[91] Park JH Rivière I Gonen M Wang X Sénéchal B Curran KJ . Long-term follow-up of CD19 CAR therapy in acute lymphoblastic leukemia. N Engl J Med (2018) 378(5):449–59. doi: 10.1056/NEJMoa1709919 PMC6637939 29385376

[92] Neelapu SS Locke FL Bartlett NL Lekakis LJ Miklos DB Jacobson CA . Axicabtagene ciloleucel CAR T-cell therapy in refractory Large b-cell lymphoma. N Engl J Med (2017) 377(26):2531–44. doi: 10.1056/NEJMoa1707447 PMC5882485 29226797

[93] Ali SA Shi V Maric I Wang M Stroncek DF Rose JJ . T Cells expressing an anti-b-cell maturation antigen chimeric antigen receptor cause remissions of multiple myeloma. Blood (2016) 128(13):1688–700. doi: 10.1182/blood-2016-04-711903 PMC5043125 27412889

[94] Brudno JN Maric I Hartman SD Rose JJ Wang M Lam N . T Cells genetically modified to express an anti-B-Cell maturation antigen chimeric antigen receptor cause remissions of poor-prognosis relapsed multiple myeloma. J Clin Oncol (2018) 36(22):2267–80. doi: 10.1200/jco.2018.77.8084 PMC6067798 29812997

[95] Ramos CA Ballard B Zhang H Dakhova O Gee AP Mei Z . Clinical and immunological responses after CD30-specific chimeric antigen receptor-redirected lymphocytes. J Clin Invest (2017) 127(9):3462–71. doi: 10.1172/jci94306 PMC5669573 28805662

[96] Topp MS Gökbuget N Stein AS Zugmaier G O’Brien S Bargou RC . Safety and activity of blinatumomab for adult patients with relapsed or refractory b-precursor acute lymphoblastic leukaemia: a multicentre, single-arm, phase 2 study. Lancet Oncol (2015) 16(1):57–66. doi: 10.1016/s1470-2045(14)71170-2 25524800

[97] Bachanova V Sarhan D DeFor TE Cooley S Panoskaltsis-Mortari A Blazar BR . Haploidentical natural killer cells induce remissions in non-Hodgkin lymphoma patients with low levels of immune-suppressor cells. Cancer Immunol Immunother (2018) 67(3):483–94. doi: 10.1007/s00262-017-2100-1 PMC6055922 29218366

[98] Neelapu SS Tummala S Kebriaei P Wierda W Gutierrez C Locke FL . Chimeric antigen receptor T-cell therapy - assessment and management of toxicities. Nat Rev Clin Oncol (2018) 15(1):47–62. doi: 10.1038/nrclinonc.2017.148 28925994 PMC6733403

[99] Varadarajan I Lee DW . Management of T-cell engaging immunotherapy complications. Cancer J (2019) 25(3):223–30. doi: 10.1097/ppo.0000000000000377 31135530

[100] Maude SL Frey N Shaw PA Aplenc R Barrett DM Bunin NJ . Chimeric antigen receptor T cells for sustained remissions in leukemia. N Engl J Med (2014) 371(16):1507–17. doi: 10.1056/NEJMoa1407222 PMC4267531 25317870

[101] Teachey DT Lacey SF Shaw PA Melenhorst JJ Maude SL Frey N . Identification of predictive biomarkers for cytokine release syndrome after chimeric antigen receptor T-cell therapy for acute lymphoblastic leukemia. Cancer Discovery (2016) 6(6):664–79. doi: 10.1158/2159-8290.Cd-16-0040 PMC5448406 27076371

[102] Gofshteyn JS Shaw PA Teachey DT Grupp SA Maude S Banwell B . Neurotoxicity after CTL019 in a pediatric and young adult cohort. Ann Neurol (2018) 84(4):537–46. doi: 10.1002/ana.25315 PMC6444896 30178481

[103] Lee DW Kochenderfer JN Stetler-Stevenson M Cui YK Delbrook C Feldman SA . T Cells expressing CD19 chimeric antigen receptors for acute lymphoblastic leukaemia in children and young adults: a phase 1 dose-escalation trial. Lancet (2015) 385(9967):517–28. doi: 10.1016/s0140-6736(14)61403-3 PMC7065359 25319501

[104] Rheingold SR Chen LN Maude SL Aplenc R Barker C Barrett DM . Efficient trafficking of chimeric antigen receptor (CAR)-modified T cells to CSF and induction of durable CNS remissions in children with CNS/Combined Relapsed/Refractory ALL. Blood (2015) 126(23):3769. doi: 10.1182/blood.V126.23.3769.3769

[105] Gust J Finney OC Li D Brakke HM Hicks RM Futrell RB . Glial injury in neurotoxicity after pediatric CD19-directed chimeric antigen receptor T cell therapy. Ann Neurol (2019) 86(1):42–54. doi: 10.1002/ana.25502 31074527 PMC9375054

[106] Landry K Thomas AA . Neurological complications of CAR T cell therapy. Curr Oncol Rep (2020) 22(8):83. doi: 10.1007/s11912-020-00935-6 32607727

[107] Gust J Ponce R Liles WC Garden GA Turtle CJ . Cytokines in CAR T cell-associated neurotoxicity. Front Immunol (2020) 11:577027. doi: 10.3389/fimmu.2020.577027 33391257 PMC7772425

[108] Liu P Liu M Lyu C Lu W Cui R Wang J . Acute graft-Versus-Host disease after humanized anti-CD19-CAR T therapy in relapsed b-ALL patients after allogeneic hematopoietic stem cell transplant. Front Oncol (2020) 10:573822. doi: 10.3389/fonc.2020.573822 33117709 PMC7551306

[109] Chen C Gu YM Zhang F Zhang ZC Zhang YT He YD . Construction of PD1/CD28 chimeric-switch receptor enhances anti-tumor ability of c-met CAR-T in gastric cancer. Oncoimmunology (2021) 10(1):1901434. doi: 10.1080/2162402x.2021.1901434 33854821 PMC8018404

[110] Adachi K Kano Y Nagai T Okuyama N Sakoda Y Tamada K . IL-7 and CCL19 expression in CAR-T cells improves immune cell infiltration and CAR-T cell survival in the tumor. Nat Biotechnol (2018) 36(4):346–51. doi: 10.1038/nbt.4086 29505028

[111] Hombach AA Geumann U Günther C Hermann FG Abken H . IL7-IL12 engineered mesenchymal stem cells (MSCs) improve a CAR T cell attack against colorectal cancer cells. Cells (2020) 9(4):873. doi: 10.3390/cells9040873 32260097 PMC7226757

[112] He C Zhou Y Li Z Farooq MA Ajmal I Zhang H . Co-Expression of IL-7 improves NKG2D-based CAR T cell therapy on prostate cancer by enhancing the expansion and inhibiting the apoptosis and exhaustion. Cancers (Basel) (2020) 12(7):1969. doi: 10.3390/cancers12071969 32698361 PMC7409228

[113] Balagopal S Sasaki K Kaur P Nikolaidi M Ishihara J . Emerging approaches for preventing cytokine release syndrome in CAR-T cell therapy. J Mater Chem B (2022) 10(37):7491–511. doi: 10.1039/d2tb00592a PMC9518648 35912720

[114] Nastoupil LJ Jain MD Feng L Spiegel JY Ghobadi A Lin Y . Standard-of-Care axicabtagene ciloleucel for relapsed or refractory Large b-cell lymphoma: Results from the US lymphoma CAR T consortium. J Clin Oncol (2020) 38(27):3119–28. doi: 10.1200/jco.19.02104 PMC7499611 32401634

[115] Kochenderfer JN Dudley ME Kassim SH Somerville RP Carpenter RO Stetler-Stevenson M . Chemotherapy-refractory diffuse large b-cell lymphoma and indolent b-cell malignancies can be effectively treated with autologous T cells expressing an anti-CD19 chimeric antigen receptor. J Clin Oncol (2015) 33(6):540–9. doi: 10.1200/jco.2014.56.2025 PMC4322257 25154820

[116] Luo W Li C Zhang Y Du M Kou H Lu C . Adverse effects in hematologic malignancies treated with chimeric antigen receptor (CAR) T cell therapy: a systematic review and meta-analysis. BMC Cancer (2022) 22(1):98. doi: 10.1186/s12885-021-09102-x 35073859 PMC8785493

[117] Freitag F Maucher M Riester Z Hudecek M . New targets and technologies for CAR-T cells. Curr Opin Oncol (2020) 32(5):510–7. doi: 10.1097/cco.0000000000000653 32657796

[118] Wagner J Wickman E DeRenzo C Gottschalk S . CAR T cell therapy for solid tumors: Bright future or dark reality? Mol Ther (2020) 28(11):2320–39. doi: 10.1016/j.ymthe.2020.09.015 PMC7647674 32979309

[119] Morgan RA Yang JC Kitano M Dudley ME Laurencot CM Rosenberg SA . Case report of a serious adverse event following the administration of T cells transduced with a chimeric antigen receptor recognizing ERBB2. Mol Ther (2010) 18(4):843–51. doi: 10.1038/mt.2010.24 PMC2862534 20179677

[120] Freites-Martinez A Santana N Arias-Santiago S Viera A . Using the common terminology criteria for adverse events (CTCAE - version 5.0) to evaluate the severity of adverse events of anticancer therapies. Actas Dermosifiliogr (Engl Ed) (2021) 112(1):90–2. doi: 10.1016/j.ad.2019.05.009 32891586

[121] Adkins S . CAR T-cell therapy: Adverse events and management. J Adv Pract Oncol (2019) 10(Suppl 3):21–8. doi: 10.6004/jadpro.2019.10.4.11 PMC7521123 33520343

[122] Davila ML Riviere I Wang X Bartido S Park J Curran K . Efficacy and toxicity management of 19-28z CAR T cell therapy in b cell acute lymphoblastic leukemia. Sci Transl Med (2014) 6(224):224ra25. doi: 10.1126/scitranslmed.3008226 PMC4684949 24553386

[123] Fitzgerald JC Weiss SL Maude SL Barrett DM Lacey SF Melenhorst JJ . Cytokine release syndrome after chimeric antigen receptor T cell therapy for acute lymphoblastic leukemia. Crit Care Med (2017) 45(2):e124-e131. doi: 10.1097/CCM.0000000000002053 27632680 PMC5452983

[124] Liu H Ma Y Yang C Xia S Pan Q Zhao H . Severe delayed pulmonary toxicity following PD-L1-specific CAR-T cell therapy for non-small cell lung cancer. Clin Transl Immunol (2020) 9(10):e1154. doi: 10.1002/cti2.1154 PMC7546952 33072320

[125] Qi K Yan Z Cheng H Chen W Wang Y Wang X . An analysis of cardiac disorders associated with chimeric antigen receptor T cell therapy in 126 patients: A single-centre retrospective study. Front Oncol (2021) 11:691064. doi: 10.3389/fonc.2021.691064 34195092 PMC8237759

[126] Rose-John S Scheller J Elson G Jones SA . Interleukin-6 biology is coordinated by membrane-bound and soluble receptors: role in inflammation and cancer. J Leukoc Biol (2006) 80(2):227–36. doi: 10.1189/jlb.1105674 16707558

[127] Fried S Avigdor A Bielorai B Meir A Besser MJ Schachter J . Early and late hematologic toxicity following CD19 CAR-T cells. Bone Marrow Transplant (2019) 54(10):1643–50. doi: 10.1038/s41409-019-0487-3 30809033

[128] Yakoub-Agha I Chabannon C Bader P Basak GW Bonig H Ciceri F . Management of adults and children undergoing chimeric antigen receptor T-cell therapy: best practice recommendations of the European society for blood and marrow transplantation (EBMT) and the joint accreditation committee of ISCT and EBMT (JACIE). Haematologica (2020) 105(2):297–316. doi: 10.3324/haematol.2019.229781 31753925 PMC7012497

[129] Brudno JN Kochenderfer JN . Toxicities of chimeric antigen receptor T cells: recognition and management. Blood (2016) 127(26):3321–30. doi: 10.1182/blood-2016-04-703751 PMC4929924 27207799

[130] Azoulay E Darmon M Valade S . Acute life-threatening toxicity from CAR T-cell therapy. Intensive Care Med (2020) 46(9):1723–6. doi: 10.1007/s00134-020-06193-1 32705293

[131] Santomasso B Bachier C Westin J Rezvani K Shpall EJ . The other side of CAR T-cell therapy: Cytokine release syndrome, neurologic toxicity, and financial burden. Am Soc Clin Oncol Educ Book (2019) 39:433–44. doi: 10.1200/edbk_238691 31099694

[132] Neill L Rees J Roddie C . Neurotoxicity-CAR T-cell therapy: what the neurologist needs to know. Pract Neurol (2020) 20(4):285–93. doi: 10.1136/practneurol-2020-002550 32503897

[133] Hunter BD Jacobson CA . CAR T-cell associated neurotoxicity: Mechanisms, clinicopathologic correlates, and future directions. J Natl Cancer Inst (2019) 111(7):646–54. doi: 10.1093/jnci/djz017 30753567

[134] Huang R Li X He Y Zhu W Gao L Liu Y . Recent advances in CAR-T cell engineering. J Hematol Oncol (2020) 13(1):86. doi: 10.1186/s13045-020-00910-5 32616000 PMC7333410

[135] Haslauer T Greil R Zaborsky N Geisberger R . CAR T-cell therapy in hematological malignancies. Int J Mol Sci (2021) 22(16):8996. doi: 10.3390/ijms22168996 34445701 PMC8396650

[136] Han D Xu Z Zhuang Y Ye Z Qian Q . Current progress in CAR-T cell therapy for hematological malignancies. J Cancer (2021) 12(2):326–34. doi: 10.7150/jca.48976 PMC7738987 33391429

[137] Larson RC Maus MV . Recent advances and discoveries in the mechanisms and functions of CAR T cells. Nat Rev Cancer (2021) 21(3):145–61. doi: 10.1038/s41568-020-00323-z PMC8353572 33483715

[138] Rahman MM Behl T Islam MR Alam MN Islam MM Albarrati A . Emerging management approach for the adverse events of immunotherapy of cancer. Molecules (2022) 27(12):3798. doi: 10.3390/molecules27123798 35744922 PMC9227460

[139] Zhao Z Li Y Liu W Li X . Engineered IL-7 receptor enhances the therapeutic effect of AXL-CAR-T cells on triple-negative breast cancer. BioMed Res Int (2020) 2020:4795171. doi: 10.1155/2020/4795171 31998790 PMC6970498

[140] Shum T Omer B Tashiro H Kruse RL Wagner DL Parikh K . Constitutive signaling from an engineered IL7 receptor promotes durable tumor elimination by tumor-redirected T cells. Cancer Discovery (2017) 7(11):1238–47. doi: 10.1158/2159-8290.Cd-17-0538 PMC5669830 28830878

[141] Pabst T Joncourt R Shumilov E Heini A Wiedemann G Legros M . Analysis of IL-6 serum levels and CAR T cell-specific digital PCR in the context of cytokine release syndrome. Exp Hematol (2020) 88:7–14.e3. doi: 10.1016/j.exphem.2020.07.003 32673688

[142] Zettler ME Feinberg BA Phillips EG Jr. Klink AJ Mehta S Gajra A . Real-world adverse events associated with CAR T-cell therapy among adults age ≥ 65 years. J Geriatr Oncol (2021) 12(2):239–42. doi: 10.1016/j.jgo.2020.07.006 32798213

[143] Lei W Xie M Jiang Q Xu N Li P Liang A . Treatment-related adverse events of chimeric antigen receptor T-cell (CAR T) in clinical trials: A systematic review and meta-analysis. Cancers (Basel) (2021) 13(15):3912. doi: 10.3390/cancers13153912 34359816 PMC8345443

